# Current Perspective on Human Milk Derived Vesicles and Their Potential Therapeutic Use: A Scoping Review

**DOI:** 10.1002/jev2.70287

**Published:** 2026-05-15

**Authors:** Patricia Hrasnova, Cristian‐Tudor Matea, Petra Gombos, Gabriel Torbahn, Andreas Marl, Marco Ginzel, Melanie Gsöllpointner, Mario Gimona, Eva Rohde, Nadja Haiden, Martijn van Herwijnen, Marca Wauben, Daniel Weghuber, Nicole Meisner‐Kober

**Affiliations:** ^1^ Department of Biosciences and Medical Biology Paris Lodron University of Salzburg Salzburg Austria; ^2^ Ludwig Boltzmann Institute for Nanovesicular Precision Medicine Paris Lodron University of Salzburg Salzburg Austria; ^3^ Department of Pediatrics Paracelsus Medical University Salzburg Austria; ^4^ Department of Pediatrics Paracelsus Medical University, Klinikum Nürnberg, Universitätsklinik der Paracelsus Medizinischen Privatuniversität Nürnberg Nuremberg Bavaria Germany; ^5^ Department of Neonatology Kepler University Hospital Linz Austria; ^6^ Medical University of Vienna Vienna Austria; ^7^ GMP Unit Paracelsus Medical University Salzburg Austria; ^8^ Department of Transfusion Medicine Paracelsus Medical University Salzburg Austria; ^9^ Department of Biomolecular Health Sciences Utrecht University Utrecht Netherlands

## Abstract

Human milk‐derived extracellular vesicles (HMEVs) recently gained significant attention due to their role in mother‐child communication and biomedical application potential. We here provide a systematic scoping review together with interactive figures and comprehensive overview tables summarising the current published knowledge base on HMEVs with a particular perspective on therapeutically relevant aspects, including consensus and divergence of isolation, characterisation, and investigation in preclinical models. Extraction from Embase and MEDLINE and systematic stratification yielded 113 original articles for further analysis. Key themes and emerging issues that became evident included (1) need for more systematic investigation and documentation of basic but essential aspects such as storage, processing parameters and endotoxin levels. (2) Changes of EV concentrations, composition, and potentially also biological activities with gestational age at birth, lactation stage and maternal background need to be taken into consideration to (3) define relevant donor selection criteria. For clinical translation, (4) the definition of mechanisms of action and relevant safety parameters is still lacking while imperative to define critical quality attributes. Eventually, (5) the availability of validated disease models to evaluate therapeutic potency represents a particular challenge for HMEVs since therapeutic approaches inherently focus on infant physiology which is difficult to model in rodents.

## 1 Introduction

Extracellular vesicles (EVs) are lipid bilayer enclosed particles typically in the nano‐ to micrometre size range that are actively secreted by living cells. This lipid bilayer allows to both, present diverse macromolecules on the surface but also encapsulate molecular components and protect them from degradation (Ngo et al. 2025, Tassoni et al. 2025). Their high stability and small size together with their built‐in functions for specific molecular interactions makes EVs ideal transport and cellular communication vehicles; they can deliver their cargo across biological barriers and influence fate and function of recipient cells and tissues even at a distance within an organism or across organismal barriers (Zeng et al. 2024, Alvarez‐Erviti et al. 2011, Kooijmans et al. 2021).

Since cells in all organisms generally produce EVs, they can be isolated from different sources, including body fluids such as blood, plasma, urine, saliva, tears, cerebrospinal fluid, or milk (Liangsupree et al. 2021, Veerman et al. 2021, Tran et al. 2025). Milk EVs have gained increasing attention since they were suggested to withstand and protect their cargo (i.e. miRNAs or mRNAs) in the highly acidic and enzyme rich environment of the stomach (Liao et al. 2017), be orally bioavailable (Khanam et al. 2023) and contribute to multiple physiological processes in mammals (Yanez‐Mo et al. 2015, Yates et al. 2022). In recent years, increasing research has focused on human milk EVs (HMEV) in particular due to their immunomodulatory effects, and their potential use for treating inflammatory and paediatric diseases (He et al. 2021, Dong et al. 2020, Miyake et al. 2020). The body of evidence highlighting diverse molecular features, biological activities and pathophysiological roles of HMEVs in relation to their donor mother's constitution has been growing rapidly in the past few years, increasing the demand for a systematic overview of the current state of the art. Here, we present a comprehensive and systematic analysis of the existing literature on human milk‐derived EVs and their potential biomedical applications, with a particular focus on the treatment of paediatric diseases. We evaluated the current scope in the field of techniques for EV isolation, methods to confirm presence of EVs and other components in the sample, as well as their characterisation in light of the MISEV (Minimal Information for Studies of Extracellular Vesicles) guidelines (Welsh et al. 2024). Finally, we critically discuss remaining issues and perspectives for the translation of HMEV‐based drug candidates into clinical testing, such as the requirement of suitable preclinical models of human paediatric gastrointestinal diseases.

## 2 Methods

To generate this scoping review, we built our search strategy based on the methodological framework outlined by O'Reilly et al. (O'Reilly et al. 2021) and adhering to the PRISMA‐ScR reporting guidelines (Preferred Reporting Items for Systematic reviews and Meta‐Analyses extension for Scoping Reviews) (Tricco et al. 2018), which is a 22‐item checklist (20 essential, 2 optional) designed to improve the reporting quality and systematic structuring of scoping reviews and ensures comprehensive and transparent documentation of methods and results. The Embase and MEDLINE databases (both via Ovid) were screened using a combination of keywords and subject heading as well as database specific syntax. All search criteria are listed in Table 1.

**TABLE 1:** Keywords used for extraction.

Embase + Classic Embase	
1	breast milk or breastmilk or breast milks or human milk or human breast milk or breast milk human or mature milk or colostrum or early milk or transitional milk).ab, kw, ti.
2	(breastfeeding or breast feeding or breast‐feeding). ab, kw, ti.
3	breast milk/
4	breast feeding/ or lactation/
5	1 or 2 or 3 or 4
6	extracellular vesicle* or extracellular membrane vesicle* or membrane vesicle* or microparticle* or microvesicle* or exosome* or ectosome* or shedding vesicle* or membrane particle* or secretory vesicle* or cell‐derived microparticle* or nanovesicle* or exovesicle* or tolerosome* or shedding microvesicle*).ab, kw, ti.
7	exosome/
8	membrane vesicle/
9	nanoparticle/
10	6 or 7 or 8 or 9
11	5 and 10

OVID MEDLINE	
1	(breast milk or breastmilk or breast milks or human milk or human breast milk or breast milk human or mature milk or colostrum or early milk or transitional milk).ab, kf, ti.
2	(breastfeeding or breast feeding or breast‐feeding).ab, kf, ti.
3	milk, human/
4	breast feeding/
5	lactation/
6	1 or 2 or 3 or 4 or 5
7	(extracellular vesicle* or extracellular membrane vesicle* or membrane vesicle* or microparticle* or microvesicle* or exosome* or ectosome* or shedding vesicle* or membrane particle* or secretory vesicle* or cell‐derived microparticle* or nanovesicle* or exovesicle* or tolerosome* or shedding microvesicle*).ab, kf, ti.
8	Nanoparticles/ or Exosomes/
9	7 or 8
10	6 and 9

Furthermore, we also searched the clinical trial registries clinicaltrials.gov, the International Clinical Trials Registry Platform (ICTRP), the Clinical Trial Information System (CTIS) as well as the European Union Drug Regulating Authorities Clinical Trials platform (EudraCT) using the keyword ‘extracellular vesicles’ and ‘exosomes’ and subjected the results to the same in‐ and exclusion criteria as for the journal articles (Supplementary File 1). For de‐duplication of retrieved articles we used the web‐based software Systematic Review Accelerator (Clark et al. 2020).

**FIGURE 1:**
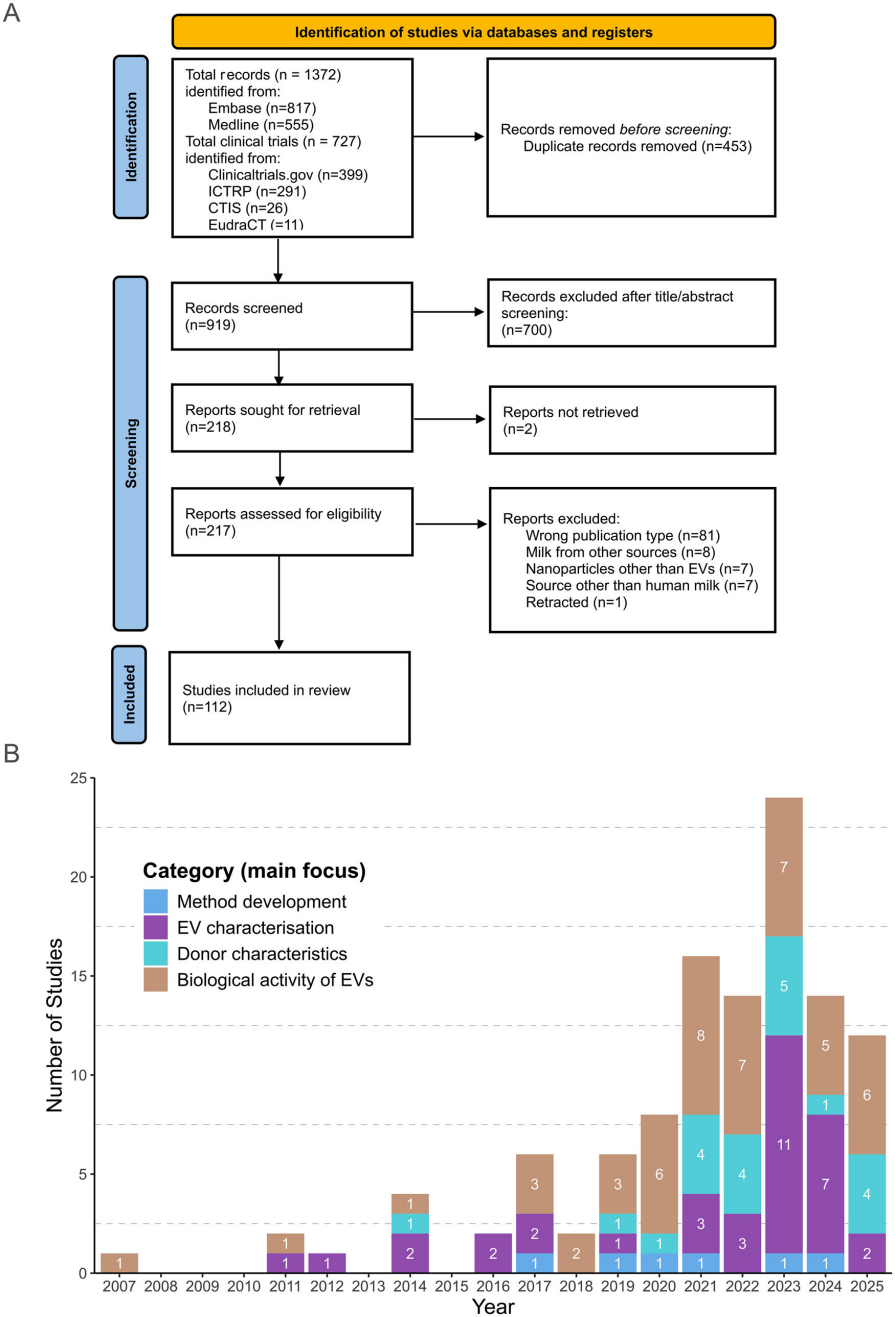
**Systematic identification and analysis of HMEV articles for this Scoping Review. (A)** PRISMA‐ScR flowchart describing the extraction and stratification of articles to be included, starting with initial identification by extraction from different scientific literature data bases using the keywords listed in Table 1, followed by automatic deduplication and manual screening by two independent scientists, resulting in 113 studies finally included in the analysis. **(B)** Articles were clustered into four major themes based on their primary focus and the numbers of articles for each category are shown in relation to publication year.

Titles, abstracts and full texts were then screened using the online version of the Covidence software which is continuously updated (Covidence systematic review software, Veritas Health Innovation, Melbourne, Australia; www.covidence.org; version August 2024). Before including the articles for the final narrative synthesis, the list of published articles and clinical trials was manually screened to remove any potential duplicates, as well as all references not meeting our pre‐specified inclusion criteria. We additionally excluded any papers that did not contain original data and/or were not peer reviewed (such as preprint articles, letters to the editor, review articles, editorials etc). Furthermore, references, that did not truly focus on human milk EVs by reporting either their characteristics and/or an experimental investigation of their biological activities were also excluded, as well as articles focusing on milk from other sources than human or on nanoparticles other than EVs (see also Supplementary File 1). The PRISMA‐flowchart for the article selection process is shown in Figure 1A.

For further stratification and final selection for inclusion into this review, the list of published articles and clinical trials was manually screened to remove any potential duplicates, any papers that did not contain original data and/or were not peer reviewed (such as preprint articles, letters to the editor, review articles, editorials etc). Finally, the content was reviewed in depth by three independent reviewers (PH, PG, CTM) to include only those articles which truly focused on human milk EVs by reporting either their characteristics and/or an experimental investigation of their biological activities. Articles focusing on milk from other sources or nanoparticles other than EVs were manually excluded. The flowchart for article extraction and stratification including the respective numbers is shown in Figure 1A.

## 3 Results

Our search resulted in 1825 titles (status 27 May 2025), of which 453 were automatically identified as duplicates. For further stratification of which articles to include, three reviewers (PH, PG and CTM) independently assessed the titles, abstracts, and full texts of the remaining 919 articles. In the next step, 701 articles which did not contain original data and/or were not peer reviewed (such as preprint articles, letters to the editor, review articles, editorials, reports of conference abstract, etc.) were manually removed. The remaining 219 research articles were subjected to full‐text review and stratified by further excluding any articles not truly focussing on human milk EVs but e.g. on milk EVs from other species, or nanoparticles other than vesicles. As a result, 116 studies were finally included into the analysis. Three of these papers had to be excluded since they were not accessible in full text; the study by Qian X. et al. (Qian et al. 2023) comparing different methods for HMEV isolation was retracted in 2024, the studies by Verma P. et al. (Verma et al. 2022) and by Jiang C. (Jiang et al. 2023) were not retrievable from the MicroRNA repository and Chinese Journal of Perinatal Medicine, respectively and also not obtained by the time of completing this review after contacting the authors. This resulted in a final number of 113 articles which were obtained fully and used for this systematic review. The flow chart of the selection process is summarised in Figure 1A, an overview of all articles that were finally included is provided in Table S1. While the first study dated to 2007, it is noteworthy that there was a marked increase in documented research on HMEVs in the past 4–5 years, with 88/113^1^ published papers after 2020 (Figure 1B; publication peak in 2023 likely due to the pandemic). We additionally clustered the studies into four categories based on their primary focus: EV isolation method, molecular characteristics of EVs, donor criteria that affect physicochemical properties and/or molecular composition of EVs, and biological activity of human milk EVs in relation to therapeutic use. As shown in Figure 1B, while the overall number of publications on HMEVs has increased in particular in the last 5–7 years, there is no obvious trend in a shift between these focus areas, indicating that all aspects of HMEVs are still warranting further investigation. However, given the still modest number of HMEV studies published, this conclusion may not reflect the current situation in the field and trends potentially already emerging now may only become apparent in published literature within the next two to three years.

In addition to the bibliographical databases, we also searched clinical trial databases using the keyword ‘extracellular vesicles’/ ‘exosomes’ followed by manual screening for trials including HMEVs. This search identified 399 entries from clinicaltrials.gov and 291 from the WHO International Clinical Trials Registry Platform, 26 from CTIS and 11 from EudraCT, however, only two of them included the use of HMEVs when the keyword ‘exosomes’ was entered at the time of extraction (27 June 2025). Both are observational studies, one targets the role of HMEVs in predicting the severity of neonatal jaundice (NCT06502847) and the other targets the role of a specific miRNA, miR‐486‐5p, present in HMEVs, and its effect on the regulation of skeletal muscle growth (ChiCTR2100052827). Neither of the studies has yet been completed.

**FIGURE 2:**
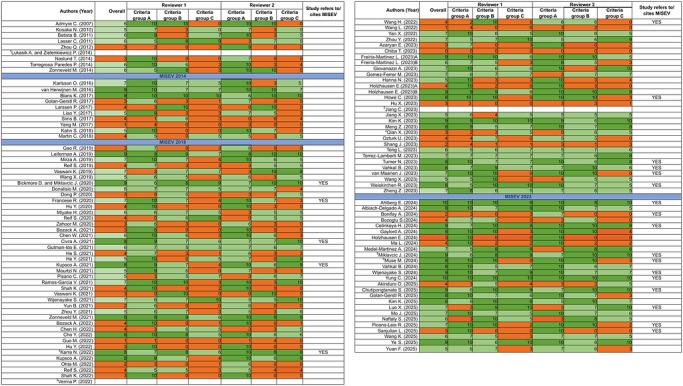
**Adherence of HMEV studies to MISEV criteria**. All articles were assessed in relation to their adherence to MISEV2023 criteria and attributed a score from 1–10 within each of three different criteria groups (Criteria group A: reporting of sample characteristics, processing and storage; criteria group B: EV characterisation by molecular composition; criteria group C: EV quantification). These assessments were first performed independently by two scientists (PH, CTM). Any remaining disagreements were discussed together with a third expert (NMK) and resolved through consensus. Scores 10–8: All or most of the criteria were met (green); Scores 7‐5: Majority of the criteria were met (light green); Scores 4–1: Most of the criteria were not met, article lacks relevant aspects for EV characterisation and/or transparent reporting based on MISEV2023. All articles were evaluated in relation to MISEV2023 guidelines but are listed in chronological order to illustrate progress over time and impact of different MISEV releases. ^1^In silico study,^2^study was retracted, ^3^study was not retrieved, ^4,5,6^studies re‐used EVs reported in a previous publication (^4^ (Zonneveld et al. 2021), ^5^ (Bickmore and Miklavcic 2020), ^6^ (Muse et al. 2024)).

To generate an evidence map on HMEVs as potential therapeutics, we structured the analysis based on the following aspects: (1) Overall adherence to MISEV criteria, (2) Collection, pre‐processing and storage of human milk, (3) HMEV isolation methods, (4) HMEV physicochemical characteristics and molecular composition, (5) impact of storage conditions and (6) donor‐to‐donor variability. Although several articles were published before the first guidelines were formulated (MISEV2014) (Lötvall et al. 2014) or updated (MISEV2018) (Théry et al. 2018), we decided to perform the analysis of all articles according to the latest release (MISEV2023) (Welsh et al. 2024), however we rank ordered them based on publication date, highlighting which MISEV versions had been published at the time of publication (Figure 2).

Finally, we (7) evaluated the characterisation of therapeutically relevant biological activities of HMEVs in preclinical disease models, including cellular, organoid and animal models. We also included a specific discussion of articles that focus on HMEVs in relation to treatment of gastrointestinal diseases with special emphasis on paediatric applications. The main conclusions from all articles were then collectively evaluated in relation to the translational perspective.

### 3.1 Adherence to MISEV Criteria

Since the establishment of the International Society for Extracellular Vesicles, the EV research community has invested much effort on ensuring rigor and standardisation for EV characterisation, quality control and reporting. The first effort was made with the publication of the Minimal Information for Studies of Extracellular Vesicles (MISEV) in 2014, which has since been repeatedly updated and refined. The most recent update to these guidelines, including for the first time also a short section on milk EVs, (MISEV2023) has introduced further aspects, including an updated summary of the consensus on markers for EVs, specific subpopulations and non‐vesicular co‐purifying components, providing guidance for more detailed descriptions of EV preparations based on the recognition of relevant parameters, and recommendations for improving characterisation at all levels.

**FIGURE 3:**
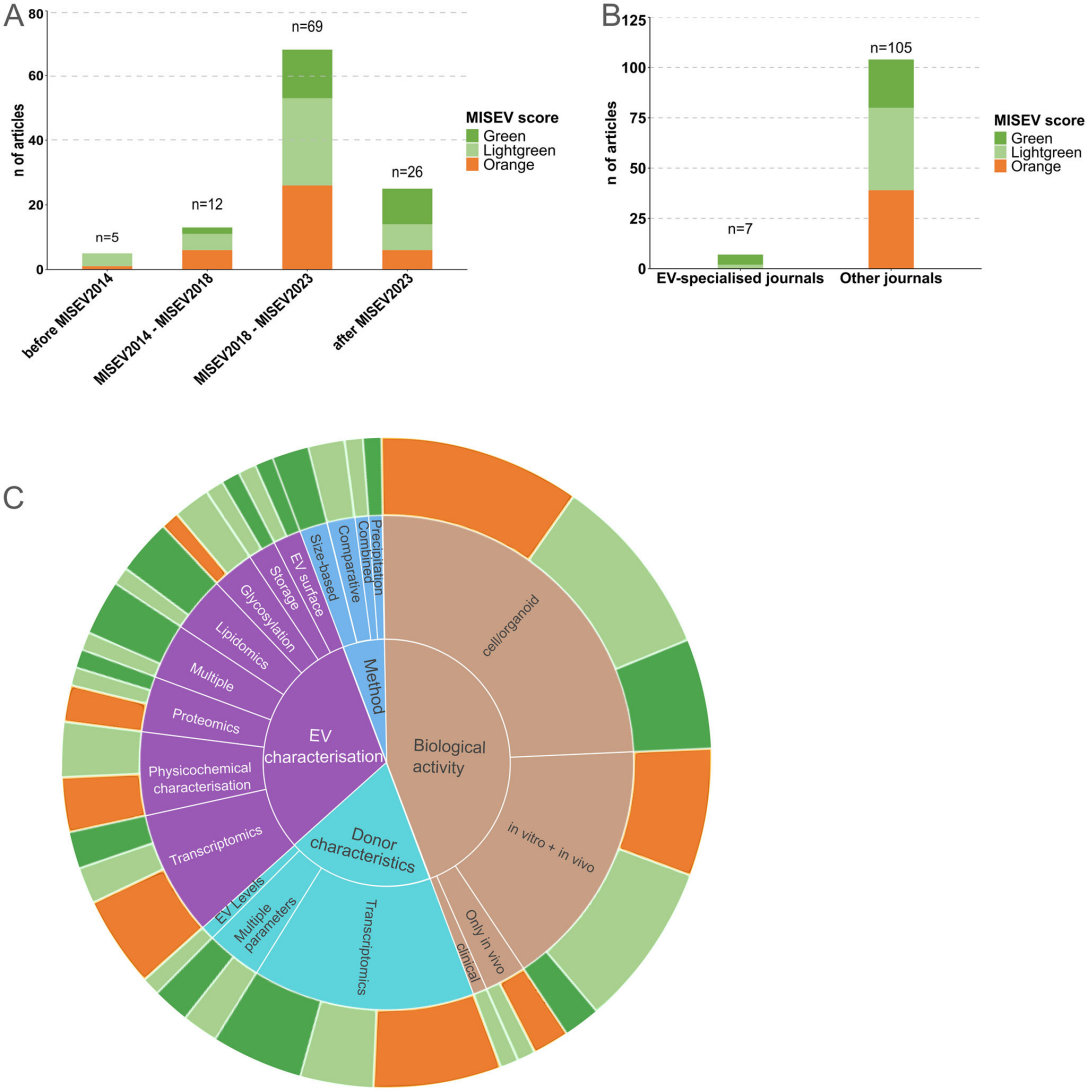
**Distribution of MISEV scores for HMEV articles** across different publication periods **(A)**, journal types **(B)** and primary research topics **(C)**. Total MISEV scores, averaged across all articles in each category, are indicated in the outer circle, with colour coding as in Figure 2 (dark green: 10‐8; light green: 7‐5; orange: below 4).

In this systematic review, we critically assessed each included research article based on three main groups of MISEV criteria: characterisation of raw material and pre‐processing (Criteria group A), EV and non‐EV markers (Criteria group B), and EV quantification (Criteria group C). In criteria group A, the focus was on the description of donor characteristics, milk handling and its storage conditions. In criteria group B, at least one protein from each MISEV category ((I) Transmembrane or GPI‐anchored proteins associated to plasma membrane and endosomes, (II) Cytosolic proteins recovered in EVs, (III) Major components of non‐EV co‐isolated structures) had to be characterised. For criteria group C, we examined whether the studies included quantitative analysis of the samples with respect to both, particle and protein content as well as information allowing to extract information on protein/particle ratios. For each of these topics, the adherence to MISEV2023 guidelines with respect to reporting details of both methods and results, as well as the parameters of experimental sample characterisation were evaluated and numerically scored. Key criteria are summarised and detailed in table S1. This was done independently by two scientists with expertise in milk EV research (PH, CTM). Any case of dispute was resolved by consensus. Articles were colour‐coded within each criteria group based on their adherence to the MISEV criteria: ‘green’ represented articles that met most or all requirements, ‘light green’ largely meeting MISEV guidelines with minor shortcomings whereas articles lacking a substantial number of characterisation parameters are grouped within the ‘orange’ category. The majority of the articles were published before the release of MISEV2023. Nevertheless, to enable the interpretation of the published data in line with the most current understanding within the EV field, we decided to assess the articles using the MISEV guidelines from 2023 rather than the respective MISEV version available at the date of publication. This may explain why a relatively large number of studies do not comply well with MISEV2023 requirements. In summary, of the 113 articles reviewed, 31 were categorised as green^2^, 42 as light green^3^, and 39 as orange^4^. One article was not classified since it was exclusively based on in‐silico data (Lukasik and Zielenkiewicz 2014). The overall scoring for all individual articles is shown in Figure 2, the detailed evaluation is provided in Table S1. Displaying the MISEV scores across publication year (Figure 3A) directly highlights the progress in the field over time, as well as the impact of the different MISEV guideline releases on reporting standards also in the HMEV field. Interestingly, the MISEV scores of publications in EV‐specialised journals were generally higher than those published in more general journals (Figure 3B), suggesting that there is a higher awareness for the requirements of adherence to MISEV criteria for EV characterisation, quality control and reporting by scientific reviewers and editors linked to the EV field. Similarly, those studies (22 in total; Figure 2, right column) which explicitly referred to the MISEV guidelines had also higher scores in meeting MISEV criteria. Figure 3c additionally shows how the adherence to MISEV criteria varies between the four focus themes of Figure 1B, revealing that publications focussing explicitly on method development have a consistently high MISEV score. In contrast, studies that primarily investigate biological activities of HMEVs are overall still lagging behind in vigour of EV quality control, thereby directly demonstrating the immaturity of the field in this aspect.

When examining the specific criteria, we found that the primary gaps of many studies were in the detailed description of EV quantification methods and the inclusion of both, EV and non‐EV markers. Most studies focused on just one category of EV markers (primarily Tetraspanins CD9> CD63> CD81) and lacked protein characterisation for categories II and III, as outlined in Welsh et al. (Welsh et al. 2024) and previous MISEV versions. From the few studies which explicitly characterised individual co‐purifying, non‐vesicular components, most focussed on beta‐Casein, Lactoferrin and Immunoglobulins, whereas, MFG^5^ as well as endotoxin (Wijenayake et al. 2021, Zonneveld et al. 2021, Wijenayake et al. 2024) were only addressed in few studies so far. In contrast to other sections in the EV field, intracellular factors originally classified as negative markers in MISEV2014 (e.g. Calnexin) are so far less generally included for characterisation of human milk EV samples. Analysing such markers may however generate additional relevant information since apoptotic bodies (both, naturally occurring as well as generated during sample handling) or ‘cell debris’ may also comprised within HMEV samples; in addition, the presence of bacterial as well as food components in whole milk and their potential contribution to the molecular profile—as well as a contribution of intact vesicles—present in final HMEV samples should not be underestimated. A clear position on the relevance of human milk oligosaccharides (HMO) content in HMEV samples is so far lacking; since it is not clear whether and which HMOs might in fact be integral components of HMEVs they can currently not be categorised as contaminant *per se*, and further investigation would be valuable to determine how HMEVs may contribute to well documented physiological activities of HMOs during nursing.

### 3.2 Collection, Pre‐Processing and Storage of Human Milk

Most studies collected milk from overall healthy, non‐drinking / non‐smoking mothers without any (documented) pre‐existing condition. Only a few studies focused on a more specific group of donors, such as from patients enrolled in a clinical study who were undergoing regular check‐ups at the hospital or were diagnosed with gestational diabetes (Shah et al. 2022), suffering from seasonal allergies (Bozack et al. 2022) or being overweight (Shah et al. 2021). Additionally, some of the milk was provided by human milk biobanks without any description of the physiological status. Almost a fifth (22/113)^6^ of the studies used colostrum as a starting material for EV isolation. We noted that the collection period for colostrum samples was generally loosely defined, ranging from as early as 1 day to 1 week after delivery^7^. Specifically, one study referred to ‘early milk’ collected from days 3–8 (Gutzeit et al. 2014) while another study (He et al. 2021) defined ‘colostrum’ as being collected up to day 3 postpartum (p.p.) and ‘milk’ collected between days 3–10, and some studies did not specify the collection period at all^8^. One group used commercially available EVs from an industrial provider/supplier (Chen et al. 2022, Chiba and Maeda 2023). A small number of studies described the collection period within specific lactation stages, such as 1 month (Golan‐Gerstl et al. 2017), 3–15 months (Zonneveld et al. 2021) or up to 6 months after start of lactation (Bickmore and Miklavcic 2020). Therefore, the description of the collection period varied in terms of terminology (colostrum or mature milk and milk from different lactation periods), highlighting that the HMEV field would benefit from closer interaction with and clear recommendations by paediatric and nutritional experts. Based on input from paediatric clinicians in our consortium and in alignment with previous meta‐analysis studies on human milk components (Thum et al. 2022), we classified the samples used in the HMEV studies included within this review as follows: colostrum up to 7 days p.p., transitional milk up to 15 days p.p. and mature milk from 15 days onwards.

Interestingly, most studies included a prospective collection of human milk samples. Only six papers explicitly reported to use milk from a human milk bank^9^, and several did not specify the origin at all^10^.The most frequently reported method for milk collection was via a manual/electric breast pump; however, almost half of the studies did not specify the exact collection method. After collection, the milk was either stored cooled or frozen, directly processed, or used without detailing the cooling and storage process. For storage of milk prior to EV isolation, most samples were kept at either −20°C^11^ or −80°C^12^. Interestingly however, when focussing specifically on articles published over the past 12 months, we noted that freezing at −80°C appears to emerge as a consensus condition for storage of human milk for later EV isolation while lower temperatures seem to be used less frequently. In most of these cases, the milk was frozen directly. A few studies first cooled the milk to 4°C before freezing at −80°C^13^, or in some cases, a pre‐processing step to skim the milk and deplete cells present in the milk by centrifugation prior to freezing (at −80°C) was included^14^. This is an important aspect to consider since it has been shown that freezing whole milk can impact the number and subpopulations of HMEVs that are subsequently isolated—potentially also influenced by the continued presence of cells after thawing (Zonneveld et al. 2014). Therefore, specific conditions for pre‐freezing of human milk still and their impact on HMEVs still require to be fully defined. Another issue that requires more detailed investigation is whether and how pasteurisation of the milk may influence the types, composition and integrity of HMEVs isolated. While two studies (Miyake et al. 2020, Torrez Lamberti et al. 2023) mentioned that the milk was processed by holder pasteurisation before EV isolation, a side‐by‐side investigation of the effect of pasteurisation would be important to refine future criteria for human milk sample collection and biobanking compatible with HMEV research.

### 3.3 HMEV Isolation Methods

In general, isolation of EVs from milk plasma is carried out by first removing or depleting other macromolecular components including casein micelles, immunoglobulins and whey proteins, as well as residual milk fat globules, while retaining and finally concentrating the vesicles. As for many other EV sources, also for HMEVs we observed a large variability in the reported isolation methods in the studies we reviewed. The most frequently used method was (differential) ultracentrifugation (UC) (53/113 studies)^15^, followed by precipitation‐based kits: ExoQuick‐TC, exoEasy Maxi, Total Exosome Isolation kit, EX04 Exo‐spin or EXOCIB kit (33/113)^16^. Seven studies combined UC and sucrose/iodixanol gradient^17^, ten studies^18^ used a combination of UC and size exclusion chromatography (SEC) and four studies used UC with a sucrose/iodixanol gradient and SEC (Zonneveld et al. 2021, Karra et al. 2022, Giovanazzi et al. 2023, van Maanen et al. 2023). We identified two studies (Chutipongtanate et al. 2022, Hu et al. 2022) employing a purely SEC approach and one study (Kosaka et al. 2010) using affinity‐based isolation with magnetic beads coated with an anti‐CD63 antibody. Three recently published studies used qEV size exclusion columns for EV isolation (Kim et al. 2023, Bonifay et al. 2024, Cetinkaya et al. 2024) following a trend of increased use of IZON columns for purely SEC or combined UC+SEC approaches for cow milk EV isolation in recent years. The most commonly used precipitation kit found in the listed studies was Exoquick (System Biosciences) (14/33)^19^, followed by the exoEasy Maxi kit (Qiagen) (9/33)^20^, and the Total Exosome isolation kit (Thermo Fisher) (5/33) (He et al. 2021, Zahoor et al. 2020, Ohta et al. 2022, Sanjulian et al. 2025, Yuan et al. 2025). These precipitation kits use hydrophilic polymers to trap the EVs and enable their co‐precipitation at low‐speed centrifugation. While this type of isolation is favourable due to the ease of use and isolation efficiency, they have limited selectivity for EVs and co‐isolate non‐vesicular components of similar size as well as residues of the polymeric structures, thereby potentially influencing the downstream results (Hendrix et al. 2023).

In addition to this wide variety of HMEV isolation strategies across the field, even within the studies using ultracentrifugation, we found significant differences in conditions, including centrifugation speeds, temperatures, times and combination of steps. In studies where HMEVs were isolated from ‘full’ milk, some protocols start with a process of low‐speed serial centrifugation steps between 3’000 – 12’000 x g^21^ for defatting, prior to HMEV isolation by ultracentrifugation without any specific steps for casein removal. A few studies combined defatting/cell debris removal with casein depletion by acid precipitation, calcium chelation by EDTA (Vaswani et al. 2019, Vaswani et al. 2021) or enzymatic coagulation by e.g. chymosin prior to UC^22^. For EV recovery, a majority of the reviewed studies employed either one or two steps of UC at centrifugational speeds ranging from 100’000 – 200’000 g^23^. Some authors specified the centrifugation conditions based on rpm (30’000 – 42’000 rpm) rather than g (Wang et al. 2019, Ramos‐Garcia et al. 2021, Zhou et al. 2022, Chen et al. 2021), whereas in most of these cases the rotor dimensions were not specified, thereby not allowing to retrieve any information on the gravitational acceleration.

Since for EV isolation, the process critically defines the sample, this lack of detail makes it hard to exactly reproduce conditions and thereby connect data between individual studies, laboratories or even operators; While this is a general issue in the EV field, for HMEVs this technical variability adds to the donor‐to‐donor variability when working with human primary material and should therefore be minimised through detailed protocols.

Interestingly, two articles used commercially available HMEVs produced by COSMO Bio (Chen et al. 2022, Chiba and Maeda 2023). According to the provider, these vesicles are produced by ultracentrifugation from a single committed^24^ donor and tested for CD9, CD63 and CD81 transmembrane protein presence. To our knowledge, this is the only published reference material for HMEVs produced commercially worldwide, unfortunately however lacking a detailed EV characterisation in line with the MISEV criteria. Therefore, while such reference materials can provide valuable tools to connect data across the field in the future, also here more detail in isolation and sample specifications will be critical.

### 3.4 HMEV Physicochemical Characteristics and Molecular Composition

We identified 35 articles^25^ that focussed on the characterisation of physicochemical properties and/or the composition of HMEVs primarily in relation to technical rather than biological questions. These included reports of either only physicochemical parameters (6/35)^26^, or an additional characterisation of the molecular composition such as RNA profiles (10/35)^27^, proteomic profiles (5/35) (He et al. 2021, Wang et al. 2022, Wang et al. 2022, Wang et al. 2025, Zonneveld et al. 2014), lipid content (4/35) (Gomez‐Ferrer et al. 2023, Albiach‐Delgado et al. 2024, Miklavcic et al. 2024, Ye et al. 2025), or glycosylation patterns/human milk oligosaccharides (3/35; HMOs) (Holzhausen et al. 2024, Batista et al. 2011, Wang et al. 2023) Additionally, there was one paper investigating the ‘surfaceome’ versus cargo proteome in a more differentiated manner (Ahlberg et al. 2024) and one study on macrogenomics/metabolomics (Wang et al. 2025). Despite recommended already in the first MISEV guidelines, physicochemical properties and/or EV protein markers were lacking altogether in 24 of the articles (Figure 2).

Most studies (18/35) isolated the vesicles using UC alone^28^ or in combination with sucrose/iodixanol gradient centrifugation (Wang et al. 2022, Wang et al. 2025, Théry et al. 2018, Vahkal et al. 2024) or SEC (Kim et al. 2023, Turner et al. 2023, van Maanen et al. 2023). Only a few used precipitation‐based isolation methods (8/35)^29^, and one study isolated EVs using affinity‐based isolation with anti‐CD63 decorated magnetic beads; thereby focusing primarily on multivesicular body (MVB) derived as well as other CD63 positive EV subsets (Kosaka et al. 2010). One of the studies used commercially available EVs from COSMO Bio (Chen et al. 2022). A recent article reported transcriptomic and proteomic data after a single HMEV isolation step using commercial SEC columns (qEV, Izon) (Cetinkaya et al. 2024).

**FIGURE 4:**
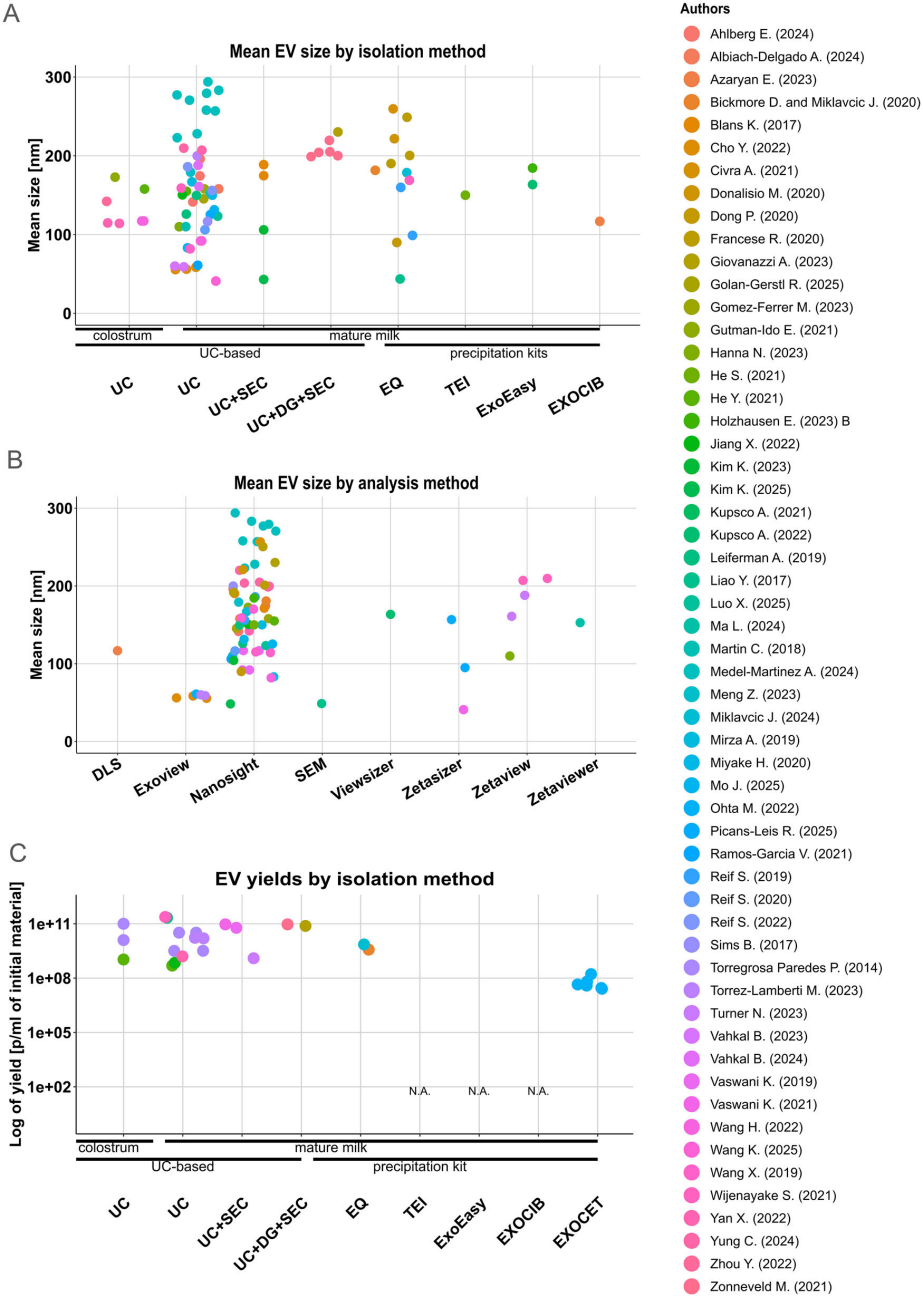
**Illustration of HMEV sizes and yields** reported across articles within this study, clustered either based on isolation method **(A and C)** or analysis method **(B)**. The corresponding articles are specified in the legend and colour coded as indicated, whereas an interactive version of this Figure is provided in Supplementary File 1 with hyperlinks from the graphs to the original articles.

Figure 4A shows the distribution of HMEV mean sizes based on the applied method. The main method used for quantifying particle size and concentration was Nanoparticle Tracking Analysis (NTA) with Nanosight instruments (Malvern) used most frequently (61/76)^30^. The Zetasizer (Malvern) was used in six^31^, and the Zetaview (Particle Metrix) in five studies (Hanna et al. 2023, Vahkal et al. 2024, Ma et al. 2024, Yung et al. 2024, Yuan et al. 2025). These measurements are all based on particle detection based on light scattering and size determination from analysis of their Brownian motion. Four articles described the application of the Exoview platform (Unchained Labs) for HMEV characterisation (Vahkal et al. 2024, Albiach‐Delgado et al. 2024, Cho et al. 2022), which is based on immuno‐capture and Single‐Particle Interferometric Reflectance Imaging Sensing (SP‐IRIS). One research group employed the ‘Exocet’ method (Ohta et al. 2022), a colorimetric approach based on acetylcholinesterase (AChE) activity for particle quantification. However, since AChE‐activity is not present in all EVs, differs substantially between EVs from different cell types and can be derived from EV co‐isolates, this method is currently not yet considered a reliable quantitation method for vesicle concentrations within the EV community.

Morphological EV characterisation, which is an integral but not mandatory parameter recommended within the MISEV guidelines, was primarily conducted using Transmission Electron Microscopy (TEM), and negative stain or cryo‐TEM imaging data are included in 60 studies (Table S1). While this method is primarily used for characterising the shape and structure, visualising electron dense components, it can additionally allow more precise and realistic sizing than solution / diffusion‐based methods. The limitations in throughput however provide a hurdle to apply it for quantification, since EV samples are generally heterogeneous and therefore a large number of particles needs to be sampled to obtain representative data for the entire population. Accordingly, only few studies use TEM as a method for size determination^32^ (Table S1).

Across all studies, the mean HMEV size ranged from 41 to 293.9 nm, whereas we would like to point out that in many cases the sizes or concentrations are specified without consideration of the significant digits obtainable by the methods thereby suggesting a measurement accuracy which is not realistic. No obvious bias by the detection method could be observed (Figure 4A), however due to overrepresentation of Nanosight‐based data this remains to be confirmed. Overall, there was a large variability between the studies even when the same isolation method (ultracentrifugation), type of milk (mature milk) and sizing methods were used (Figure 4B). Interestingly, relatively few studies reported the yield of EVs in terms of particles per mL of initial material, which would be the most direct parameter to compare yields across studies, and some studies only report total protein yields—even in recent publications (Gao et al. 2019, Turner et al. 2023, Admyre et al. 2007). A few studies reported HMEV concentrations in the final samples, however with no information on yields. The yield across all studies—wherever specified^33^—varied from 5 × 10^7^ particles/mL (Dong et al. 2020) to 9.3 × 10^10^ particles/mL (Zonneveld et al. 2021) (see Figure 4C), averaging between 1 × 10^9^ up to 1 × 10^11^ particles/mL. Even though the yields were calculated mostly based on Nanosight measurements, there were two studies that used Zetaview (Hanna et al. 2023) and ‘Exocet’ method (Ohta et al. 2022). The comparison in between these results should be therefore taken cautiously as there is no comparative study analysing side‐by‐side differences among these instruments. Due to the lack of standardisation and reporting of important details determining the LODs, however, a direct comparison of these data needs to be considered with caution.

Protein concentrations were predominantly determined by BCA‐based assays. The final concentrations varied from 0.6 to 44 mg/mL. However, since not all articles provide information on how these numbers relate to the original starting volume, and since only three of these articles specified also particle concentrations, an assessment of the variability in protein yields and protein/particle ratios across the literature cannot be made.

For characterisation of HMEV protein markers, most studies relied on western blotting, which in principle is possible for all isolation methods and mainly limited by the yields. Also, for unbiased molecular profiling (proteomics, transcriptomics, lipidomics), there is no emerging consensus on preferential HMEV isolation methods, and most commonly used protocols seem to generally be technically compatible with sample preparation for most *‐omics* technologies. Identification of HMEV surface markers by flow cytometry was so far only reported for dUC derived samples (4/113) (Hu et al. 2020, He et al. 2021, Admyre et al. 2007, Cho et al. 2022).

Reflecting that a transfer of miRNA to recipient cells has become one of the most widely hypothesised functions of EVs in general, also our analysis of the HMEV literature revealed that RNA was the predominantly investigated type of HMEV cargo so far (11/36 studies)^34^. Interestingly, with the exception of three studies on either lncRNAs (Karlsson et al. 2016), tRNA‐derived RNAs (Gaylord et al. 2024) or the plant RNA content in HMEVs (Lukasik and Zielenkiewicz 2014), all of them concentrated on miRNAs; the main questions of interest included the impact of storage conditions or lactation stage on the miRNA profile, as well as the identification of miRNA candidates of potential therapeutic relevance. As discussed above, the protocols to prepare the final EV sample varied significantly across these studies; notably, no two analyses used the same parameters (e.g. centrifugal speed in UC, precipitation kit provider, time points post‐partum or milk storage conditions). Three studies reported a correlative analysis of both the RNA and proteome profiles of HMEVs (Kim et al. 2023, Turner et al. 2023, Cetinkaya et al. 2024). These were used as a more comprehensive readout to either identify antimicrobial patterns in the HMEV samples (Kim et al. 2023), for comparing available milk formulas with human and cow milk characteristics (Turner et al. 2023) or for analysing the vesicular content after comparing different casein depletion methods (Cetinkaya et al. 2024).

Interestingly, only a few articles so far explored the glycoprotein profiles and lipid content of the HMEVs, despite the fact that both, lipids and HMOs have been extensively investigated as bioactive components of human milk (Holst et al. 2023, Schalich et al. 2024, Renwick et al. 2025). Only two articles analysed the content of polyunsaturated fatty acids of HMEVs (Gomez‐Ferrer et al. 2023, Albiach‐Delgado et al. 2024), suggesting low abundance but bias towards anti‐inflammatory oxylipins. Another study reported the HMEV fatty acid content is altered in milk from mothers with genetic diseases of fatty acid metabolism (fatty acid desaturase gene cluster, FADS) (Miklavcic et al. 2024). What still remains to be demonstrated is which of these fatty acids are integral components of the vesicles or co‐isolates within the HMEV samples. Similarly, the glycosylation profile as well as HMO content of HMEVs remains largely elusive. While an early study in 2011 (Batista et al. 2011) already provided first data on the glycan profile of HMEVs using lectin arrays, this was followed by a gap of twelve years until another publication came out in 2023 extending this to an unbiased analysis of the glycosylation pattern by glycoproteomics (Wang et al. 2023), followed by a recent report of a combined analysis of HMOs and miRNAs (Holzhausen et al. 2024). An interesting recent study reports an advanced proteomics method to differentiate HMEV ‘surfaceome’ from cargo proteome using a lipid based protein immobilisation (LPI‐) based LC‐MS strategy (Ahlberg et al. 2024), which is in line with the generally increasing interest in the EV community to better understand the composition, relevance and function of the EV corona in different biological matrices (Tassoni et al. 2025, Buzas 2022). Also, novel QC approaches using ATR‐FTIR (Ramos‐Garcia et al. 2021), or di‐electrophoresis have entered the stage for characterisation of human milk EVs (Chen et al. 2022). Whereas it remains still unclear how these readouts relate to both, the molecular composition and the biological properties of the vesicles, we note a general trend towards a more comprehensive characterisation of HMEVs combining multiple parameters and components, as well as a more differentiated profiling between surface and cargo components.

A key unresolved issue however is to interpret any correlation between vesicle function and their composition in light of the biases introduced on the (sub‐)populations of HMEVs and co‐purifying species by the isolation procedure and truly assigning cargo of interest to the vesicles. In particular, purely UC‐based methods as well as precipitation‐based kits are in general known by now to co‐isolate large fractions of non‐vesicular components, making it difficult to unambiguously assign cargo and functions to be truly associated with vesicles. This issue has been for example uncovered already a decade ago for serum EVs; while the initial hypothesis was that a large number of circulating miRNAs are linked to extracellular vesicles, it was later demonstrated that this is only true for a small fraction of specific miRNAs, whereas the vast excess of circulating miRNA is associated with non‐vesicular, Argonaut‐protein containing ribonucleoprotein (RNP) complexes (Arroyo et al. 2011). Similar findings were meanwhile reported for other EV sources (Chevillet et al. 2014) and it will be important to critically assess how suboptimal isolation methods may affect the reliability of downstream analysis results also for HMEVs. This is an important issue since one of the most studied hypotheses is that the anti‐inflammatory activities of HMEVs are largely attributed to the transfer of known immunomodulatory miRNAs (i.e. miR‐148, miR‐155, miR‐146, miR‐21, miR‐26, miR‐30). At the same time however, many of these studies were within the ‘orange’ MISEV score category as revealed in Figure 3C, lacking comprehensive EV characterisation or direct evidence for miRNAs being truly associated with the HMEVs. Given the increasing notion that a large majority of extracellular RNA in many body fluids is not associated with vesicles but is rather co‐purifying within non‐vesicular components, a lack of basic characterisation of bona fide EV versus non‐EV markers within the samples is particularly problematic. To deepen our understanding of the role of miRNAs in human milk (Bozack et al. 2021, Shah et al. 2021, Bozack et al. 2022, Shah et al. 2022), it will, however, be imperative to unambiguously resolve which miRNAs are indeed linked to EVs, or which ones are associated with non‐vesicular components, and how this relates to their biological activity. We would therefore like to point out that these limitations need to be taken into consideration for interpretation of key themes and conclusions published on the role of miRNAs or other specific molecular factors in HMEV biology so far. Also, these ambiguities highlight once more how the heterogeneity of EV samples together with variations in isolation as well as characterisation methods make standardisation and comparability of results, as well as bona fide conclusions on EV versus non‐EV related activities still highly challenging also in the human milk EV field.

### 3.5 Impact of Storage Conditions

According to our systematic analysis, Zonneveld et al. and Leiferman et al. (Leiferman et al. 2019, Zonneveld et al. 2014) published the only two articles so far that systematically investigate the impact of the storage conditions and processing steps on human milk EV recovery and/or characteristics. While Zonneveld et al. focused primarily on the initial storage conditions of human milk and their impact on the subsequently isolated vesicles, Leiferman et al. investigated the effect of storage of the HMEVs themselves on selected EV parameters (size, concentration, selected miRNA cargoes). Both studies revealed that storage conditions of both, raw milk and isolated vesicles can substantially affect recovery, yields, quality and composition of HMEVs. It is thus conceivable that also the biological activity of the vesicles might be affected, however this remains to be further investigated. Therefore, additional work to broadly and systematically investigate the impact of different storage and processing conditions on HMEV recovery, properties and activity would be of substantial value for the field. The overall composition of human and bovine milk is largely comparable, except for a lower content of total protein (in particular Casein) and Calcium, as well as a higher content of oligosaccharides and immunoglobulins in human milk (Box 1). While this does require certain optimisation of isolation protocols and has implications on potential co‐isolated components, it is conceivable that the general requirements for storage and pre‐processing are still largely overlapping. Thus, the HMEV field could benefit here from knowledge transfer from the bovine milk EV field which is more advanced in investigations of storage conditions, facilitated by the easier access and larger scales of the source material.

**BOX 1:** Composition of human milk and colostrum (Kim and Yi 2020), cow colostrum (Kehoe et al. 2007) and cow milk (Zhou et al. 2022).

Component	Human colostrum [%]	Cow colostrum [%]	Mature human milk (≥ 14 days p.p.) [%]	Cow milk [%]
**Fat**	1.5–2	6.7	3.5–4	4
**Total Protein**	1.4–1.6	14.9	1	3.1
**Sugars** **(Lactose / Oligosaccharides)**	5–6.2	2.5	6–7	5
**Ash**	—	0.05	0.2	0.7
**Total Solids**	8.1–10	27.8	10.5–12.2	12.9

Data were taken from references (Kim and Yi 2020) for human colostrum and mature milk and (Kehoe et al. 2007, Foley and Otterby 1978) for cow colostrum and cow milk, respectively. Ash: all non‐organic components, including minerals such as calcium, phosphorus, potassium, magnesium, sodium, and chloride.

*Note*: specifications for human colostrum include data only up to 5 days pp. Based on input from paediatric clinicians in our consortium, we recommend for future HMEV studies to classify the sample collection period as follows: colostrum up to 7 days p.p., transitional milk up to 15 days p.p. and mature milk from 15 days onwards.

### 3.6 Donor Parameters Influencing the Characteristics of human Milk EVs

We identified 22 studies that exclusively focussed on the impact of donor‐to‐donor variability and/or specific donor parameters on the composition, levels and physicochemical properties of HMEVs^35^, and one study that investigated the impact of gestational age and diabetes of the mother not only on HMEV properties but also their biological activity in modulating hepatocyte proliferation (Zheng et al. 2023). These studies were assigned to the ‘biological activity’ category of this review. Interestingly, a large majority of these studies concentrated on miRNA content^36^. The mothers' health conditions were the most prominent factors demonstrated so far to significantly affect miRNA profiles in isolated HMEVs. The isolation methods used for these analyses were primarily spin‐column and/or precipitation‐based commercial kits (14/22)^37^ whereas ultracentrifugation was used less frequently (6/22)^38^. As a general conclusion, both mental and physical health issues were associated with changes in miRNA profiles of HMEV samples. Psychological assessments of stress disorders during the first and second trimesters of pregnancy (Bozack et al. 2021), physical factors such as maternal overweight or obesity (Shah et al. 2021, Cho et al. 2022), metabolic disorders like gestational diabetes or type 1 diabetes (Shah et al. 2022, Zheng et al. 2023) and disorders including asthma, allergies, and viral infections like HIV‐1 or SARS‐CoV‐2 (Zahoor et al. 2020, Bozack et al. 2022, Chutipongtanate et al. 2025, Gutzeit et al. 2014) were linked to alterations in the miRNA distribution. Similar associations between HMEV miRNA profile and the mothers’ physical condition have been proposed for effects of hormonal activity (Golan‐Gerstl et al. 2017) and inflammatory bowel disease (IBD) (Golan‐Gerstl et al. 2025). Notably, specific miRNAs associated with inflammatory responses (miR‐148a‐3p, let‐7a‐5p, miR‐146b‐5p, miR‐21‐5p, miR‐26a‐5p, and miR‐30d‐5p) were repeatedly identified in HMEV samples across different and independent studies. This is generally in line with the role of human milk as a source of inflammation modifying agents and indicates that these anti‐inflammatory miRNAs are selectively retained in HMEVs from donors of diverse backgrounds. Given the issues of EV characterisation and isolation method biases discussed in section (3.4), the bona fide association of miRNAs with the vesicles themselves as well as the functional transfer of anti‐inflammatory into recipient tissues by the HMEVs however requires further substantiation.

Apart from inter‐donor variability, some studies also assessed the influence of lactation stage (Gao et al. 2019, Admyre et al. 2007, Gutzeit et al. 2014), including one study reporting changes in particle concentrations at different time points post‐partum (Ohta et al. 2022). One study (Mourtzi et al. 2021) focussing on long noncoding RNAs (lncRNAs) also reported variations based on the mother's delivery date, revealing that preterm birth appeared to significantly reduce the expression of RNAs involved in DNA damage response within HMEVs. A combined investigation of both RNA and protein analysis as a function of donor parameters was reported by Vahkal et al. (Vahkal et al. 2024) which revealed a significant influence of gestational age at birth on HMEV RNA and protein cargo. Of note, this study was one of only few that specifically included mRNAs in the RNASeq analysis.

Beyond the spectrum of pathophysiological conditions it is conceivable that also nutrition as well as environmental factors may influence the levels, composition and function of EVs in human milk; First data in this direction have been reported in interesting studies addressing very specific questions, such as the effect of exposure to marine pollutants in the Faroe Islands on HMEV miRNA profiles (Kupsco et al. 2022), or another study investigating the proteomic profile of HMEVs in relation to the mother's lifestyle and environmental factors such as air pollutants (Gutzeit et al. 2014), discussing additionally how these effects might influence the later development of allergies in the children.

In conclusion, the data reported so far indicate that the human milk EV proteome and miRNA profiles may change from colostrum to mature milk and over the lactation stage and may additionally vary between individual maternal health conditions. While much research has primarily concentrated on changes in miRNAs, a substantiation of existing data as well as a deeper and more holistic examination including the proteomic, lipidomic, metabolomic and glycan profiles should be generated in the future to address how donor parameters may affect the composition as well as biological activities of human milk EVs. Furthermore, also a potential influence of environmental factors, maternal nutrition and lifestyle remains to be elucidated.

### 3.7 Biological Activities of Human Milk EVs and Testing in Disease Models

Of the 50 articles that investigated the biological activity of HMEVs^39^, 28 investigated them as potential therapeutic agents in preclinical disease models^40^. These included EVs from various human milk sources from colostrum to mature milk, primarily isolated by ultracentrifugation (27/50)^41^. Despite the open issues of using precipitation‐based methods for the downstream investigation of EV functional activities discussed in section (3.3), several studies investigated HMEVs obtained by Exoquick precipitation in disease models^42^. Strikingly, thirteen articles focused on the therapeutic use of HMEVs in models of necrotising enterocolitis^43^, which is a clear application opportunity given that the current paediatric recommendation for prevention of NEC onset is feeding babies with donors’ milk (Maffei and Schanler 2017). Interestingly, several studies examined the potential of HMEVs for prevention or treatment of viral infections which are either typically contracted during infancy (Cytomegalovirus, Rotavirus, (Leiferman et al. 2019, Francese et al. 2020)) or result in complications during pregnancy (Zika, Usutu (Freiria‐Martinez et al. 2023)). Our analysis further showed that EVs were predominantly used in *in vitro* studies, with only a few *in vivo* studies conducted preclinically, all of which were performed in rodents so far (C57BL/6 and BALB/c mice or Sprague Dawley rats) with animal age ranging from one day to 8 weeks, and treatment periods spanning from 4 (Chen et al. 2021) to 10 days (Reif et al. 2022). To provide an overview of the data currently available on the characterisation of the biological activities of HMEVs, we defined a short list of criteria for classification such as type of model (cellular/organoid/animal), therapeutic paradigms (preventive versus treatment), type of disease, etc (Table S2). Most of the relevant articles investigated the protective properties of HMEVs in models of cellular stress, predominantly using commercially available cell lines, e.g. from publicly accessible repositories like ATCC. We also identified four studies that used either stem cells or organoids derived from primary mouse tissue (ileum) to test the effect of HMEVs (Dong et al. 2020, Miyake et al. 2020, Gao et al. 2019, Reif et al. 2022) in models of necrotising enterocolitis. A recent study for the first time described the use of human neonatal small intestinal organoids for an HMEV study, however not yet addressing any therapeutic effects but focussing on HMEV uptake (Luo et al. 2025). Our analysis further showed that in cellular models, human milk EVs were primarily explored in a therapeutic paradigm (co‐treatment and/or post‐treatment, 12/27)^44^, with fewer studies investigating prophylactic use (7/27)^45^ or comparing prophylactic and therapeutic paradigms in the same cellular model (4/27) (Donalisio et al. 2020, Francese et al. 2020, Civra et al. 2021, Reif et al. 2022). Interestingly, in most *in vivo* studies the HMEVs were applied alongside disease induction thereby making it difficult to clearly dissect preventive from therapeutic effects (Miyake et al. 2020, Pisano et al. 2020, He et al. 2021, Zhou et al. 2022, Wang et al. 2019). Except for the studies of viral infections (Donalisio et al. 2020, Francese et al. 2020, Civra et al. 2021, Naslund et al. 2014), many disease models were based single chemical or environmental stressors to trigger disease onset. In particular, rodent models for colitis or necrotising enterocolitis used detergents in co‐treatment with (human milk) EVs (He et al. 2021, Dong et al. 2020, Gao et al. 2019, Guo et al. 2022, Chen et al. 2021). While this is a standard model of colitis‐related indications, it may warrant caution for studies involving co‐treatment with EVs because of the potential disruption of vesicles by the detergent used for inducing the gastrointestinal defect.

Among all studies, the reporting of dosing of HMEVs in both cellular, but in particular *in vivo* models are still inconsistent and ambiguous, making it hard to compare studies across the literature and to predict how realistic their therapeutic application really will be. For example, a majority of the studies specified EV doses in µg to mg of proteins rather than particle numbers. This is problematic because of the content of co‐purifying non‐vesicular proteins which are known to vary with sample pre‐processing conditions, isolation methods and—in the case of HMEVs—further differ in the starting material across lactation stage. Furthermore, as is the case for many other areas in EV research as well; there is still a need for defining appropriate controls to support claims of biological activities of HMEVs. First advances were found in a few studies that either used human milk supernatant as a vehicle control (Donalisio et al. 2020, Civra et al. 2021) or treated the cells or animals additionally with EV‐depleted human milk samples (Dong et al. 2020, Karra et al. 2022). As milk has well‐described anti‐inflammatory properties associated with non‐vesicular components such as lactoferrin (Atayde et al. 2024, Wisgrill et al. 2018) or human milk oligosaccharides (HMOs), such thorough controls will be essential to unambiguously determine the therapeutically relevant biological activities of HMEVs.

## 4 Discussion

In this scoping review, we searched Embase and OVID MEDLINE databases up to 27 May 2025, and, starting from 1825 recorded on May 27^th^, 116 original articles on human milk EVs were identified by systematic literature extraction, of which 113 could be included in the analysis.

Since adherence to minimal reporting and quality control standards is an issue of particular importance in the EV field, our analysis included an extensive in‐depth assessment of the methodological details reported as well as the data provided in light of the MISEV guidelines (Figure 2). This included not only scoring based on a ‘checklist’ but an assessment by two independent experts of how well conclusions on quality control are supported by the data, and whether it would be possible for someone skilled in the art to reproduce the methodology based on the conditions specified. Thereby the scoring, while providing added scientific value beyond simple metrics, is inherently prone to some subjective assessment. To mitigate divergence in some scores, any conflicts were resolved by an in‐depth consensus discussion with a third independent expert. To make this entirely transparent, the individual as well as the consensus scores are documented in Figure 2.

In depth analysis revealed that methodological parameters used for milk EV preparations as well as characterisation and reporting differed considerably across all studies. Remarkably, no two groups used the same approach regarding the starting material, isolation process and characterisation, including choice of parameters to quantify HMEVs versus co‐isolates. In consequence, harmonising the results published so far remains complex and highlights the necessity for a minimum standard in documentation and reporting, in line with international consensus and guidelines such as MISEV. This is an even more essential issue in fields dealing with EVs from human primary sources due to the additional complexity contributed by donor‐to‐donor variability as well as potential changes across lactation stages, nutrition or other environmental factors even for the same donor. In that context, a striking notion from this review is that only 70 out of the 113 articles specified the donor parameters in depth. As evident from Figures 2 and 3, the release, regular update and continuous advocating for adherence to the MISEV guidelines starts to make a positive impact also on the HMEV research field. In turn, a gap is to be expected between early literature and recent as well as future research on HMEVs, where it will become increasingly difficult to relate new data to those from early, pioneering studies. To facilitate tracking of analytical data on EVs, ISEV has introduced EV‐TRACK, a reporting repository aligned with MISEV and designed to provide transparent and easy overview of studies on EVs (Van Deun et al. 2017). We found only 15 of the HMEV studies^46^ included in this review also integrated into the EV‐TRACK database, indicating that there is strong need for improved reporting standards and usage of international repositories in the HMEV field.

Accordingly, for the articles included in this review, remaining inconsistencies in basic aspects such as lack of comprehensive reporting, specification of HMEV concentrations and yields, as well as storage and processing methodology complicated a comprehensive comparison and overall interpretation of published data. Nevertheless, the following emerging issues and themes became evident: (1) HMEV concentrations, composition and potentially also biological activities can vary considerably based on maternal genetic background and health conditions. In addition, early data suggest that they may further change with gestational age at birth and lactation stage as well as environmental factors (such as maternal nutrition or stress). (2) While current data are mainly focussed on individual aspects such as EV levels or individual components, more holistic investigation will be needed. Within the reviewed studies, a recurrent theme was the strong focus on miRNA profiles within HMEV samples, whereas additional data on the vesicles themselves were not always included. Given that in many biofluids, cell free miRNAs are primarily not associated with EVs (Arroyo et al. 2011, Endzelins et al. 2016), it is crucial to provide unambiguous data showing true encapsulation of miRNAs within or in association with HMEVs, as well as their functional transfer into recipient cells, when making conclusions about miRNA dependent HMEV functions, such as the modulation of inflammatory responses. We would therefore like to raise caution about the possible overinterpretation of data that often relate HMEV activities primarily to miRNAs, despite lacking such unambiguous evidence. In addition, while many studies focused on the miRNA content of HMEVs, their lipidome, proteome and metabolome as well as detailed physicochemical properties are still underexplored, although all likely critical for understanding their function. This presents a need for further research on the relevance of protein, lipid, metabolite and glycan—as well as RNA—composition for the biological activity and potential therapeutic effects of HMEVs. Furthermore, there is an increasing recognition that the molecular HMEV surface composition—including proteins and factors reversibly attached and captured under the term ‘EV corona’—may be highly relevant for their function in different biological milieus. With the exception of one article that has applied LC‐MS for ‘EV surfaceome’ characterisation (Ahlberg et al. 2024), there is still a complete lack of knowledge about the corona of HMEVs. Since the complex matrix of milk may contribute substantially to the functionalisation of HMEVs, it will be important to dedicate further research to understand the interplay between the vesicles and molecular components in their environment. (3) In order to unambiguously dissect these translational questions, basic technical issues such as the impact of storage, processing and isolation conditions remain to be systematically addressed, considered and documented in future studies. One of the main observations when reviewing all articles was the wide range of different milk collection and pre‐processing parameters (storage conditions, lactation stages, donor parameters) and conditions for HMEV isolation methodology (centrifugal speed, temperature, number of cycles in UC or the provider of the precipitation kit) whenever specified. However, starting material, pre‐processing, storage as well as isolation procedure will directly impact the amount and types of EVs present and recovered as well as the co‐purification of other components such as milk fat globules, casein micelles, protein complexes and cell death related byproducts. Since *‘the process is the product’* directly applies also to HMEVs, these differences likely contribute a main factor to the substantial variations in the final samples reported. While it is not realistic that uniform protocols will ever be used across the entire field at least for basic research, this diversity emphasises the necessity for systematic characterisation and documentation of key parameters for all relevant steps, including donor parameters, milk collection and pre‐processing, HMEV preparation and storage conditions, and adherence to common guidelines such as MISEV will be essential for comparability of data. Given the complexity of the human milk matrix and the possible interplay between different milk components, including HMEVs, may warrant a refinement of MISEV guidelines specifically for HMEV research in future updates. (4) As for all other therapeutic candidates, clinical translation will critically depend on the availability of validated disease models; This represents a particular challenge in the diseases most intensely researched for human milk EVs: paediatric diseases and gastrointestinal inflammatory conditions. For infants, not only patient complexity but also organ development needs to be considered. Special attention is further required when studying gut development and inflammation, as there are notable differences between human and rodent pathophysiology. Interestingly, of the 113 reviewed articles only 50 included data on biological activities of HMEVs, which primarily focused on commercially available cell lines. Twenty‐one of these studies included also animal data, all from models using rodents. Thus, further progressing into larger animal and/or human organoid models (Yung et al. 2024, Luo et al. 2025) will likely be key to predict the application potential and scope of HMEVs as therapeutic agents or biomarkers. As a prerequisite however, further improvements are needed in order to rationally devise dosing schemes before moving into studies involving higher species; Our analysis also showed that the specification of vesicle doses across all studies was highly inconsistent and often ambiguous since EV concentrations—instead of particle doses—were mostly reported based on protein content, which is largely determined by the excess of protein from co‐purifying non‐vesicular components and additionally varies dramatically with isolation procedure and handling. The dosing schemes not only varied even between studies using similar models but sometimes additionally lacked specification of timing. Furthermore, also the routes of administration varied substantially, and so far there are no data available on quantitative tissue biodistribution and pharmacokinetics of HMEVs even in rodent models. For progressing into larger animal models and, ultimately, clinical translation, it will therefore be important to generate more robust and well documented quantification of dose‐response relationships, not only in animals but even in cellular models. Furthermore, appropriately matched, e.g. EV‐depleted controls will become key to dissect truly HMEV‐related activities from contributions of non‐vesicular components and enhance our understanding of the potentially synergistic interplay of the vesicles with other bioactive factors naturally present in milk, thereby informing on the optimal composition of HMEV therapeutics. Both, robust pharmacokinetic‐pharmacodynamic (PK/PD) data linked to specific routes of administration, as well as information on relevant HMEV sample characteristics will be essential to design treatment paradigms for study protocols in larger animals and, ultimately, patients.

Despite these limitations, first data for human milk EVs in disease related cellular and rodent models are promising. Since the molecular mechanisms of action still remain largely elusive, at least a basic understanding of functionally relevant features or components of the HMEVs will be essential to define critical quality attributes (CQAs) required for clinical translation. Furthermore, there is still a noticeable lack of data that specifically address potential adverse effects or dose limiting parameters. While it is generally considered that potential immunogenicity may be less relevant for HMEVs than for cross‐species applications, this hypothesis still remains to be corroborated. However, we would like to point out that except for three of the publications which either specified endotoxin levels (Zonneveld et al. 2021) or microbial content (Wijenayake et al. 2021, Wijenayake et al. 2024) even basic safety‐relevant parameters like bioburden, enrichment of detrimental impurities, or endotoxin levels are so far not specified in most studies, pointing again towards the need for better documentation and increased analytical standards in order to progress towards preclinical PK/PD and ADME‐Tox studies.

## 5 Conclusions and Recommendations for Future Directions in the HMEV Field

(1) Adherence to MISEV criteria and use of EV‐TRACK: One of the overarching conclusions from this systematic review is that the methodology for human milk collection, pre‐processing, storage as well as isolation and characterisation of HMEVs varies substantially across the studies included in this review, with no two studies across different labs following the same or comparable procedures. Since it is not realistic that the field will converge to ‘*the’* isolation and QC processes for human milk extracellular vesicles in the near future, the reporting of details and a consensus on minimal standards will be even more essential. For both there are in fact already existing guidelines (MISEV) as well as repositories (e.g. EV‐TRACK) developed with intensive efforts by experts in the field. Our analysis shows that in the HMEV field, possibly even more than in other areas of EV research, adherence to MISEV criteria (Figure 2) as well as use of EV‐TRACK (with only 15 out of 113 studies registered) is still poor, thereby missing an opportunity to connect the data across the field and draw broader conclusions beyond individual articles. Furthermore, EV‐TRACK serves as a prospective checklist for authors to meet acceptable standards for reporting and analytics. An interesting notion was that the number of HMEV articles published in non‐EV‐specialised journals was high (105 out of 113 studies), and the adherence to MISEV criteria differed remarkably between these two types of target audiences (Figure 3B). We therefore strongly advocate for *(i)* HMEV researchers to proactively upload any study into EV‐TRACK in parallel to the peer‐review publication process, which should inherently raise higher awareness for consideration of both, reporting and analytical requirements and *(ii)* Reviewers and Editors to be more aware and consequent about the requirements of minimal standards in the EV field, facilitated through guidelines such as MISEV, prior to acceptance of articles on HMEVs for publication, even or in particular if to be published in non EV‐specialised journals.
(2) The HMEV field would benefit from more extensive knowledge transfer from research on milk vesicles from other mammalian species. In particular research on cow milk vesicles is in many aspects more advanced including processes for isolation, data on characterisation as well as biological activities. Also, exciting research on other species such as in depth studies of transfer of milk vesicles from genetically engineered mice to pups during nursing (Zhou et al. 2022, Nørgård et al. 2022) is providing new insight that will ultimately also benefit the HMEV field. Given the issues of incomplete reporting and high variability in methodology, future studies providing a side‐by‐side comparison of milk EVs from different mammalian species using the same methods and assays would be of high value. In particular for therapeutic applications, a very practical key question is whether and how human milk vesicles diverge from EVs derived from e.g. dairy products; Given the additional hurdles for developing therapies from human sources rather than food derived material, it will pay off to systematically investigate whether and how milk vesicles from human origin differ in efficacy and/or safety. To enable such work, simple issues such as robust characterisation of species selectivity vs cross‐reactivity of tool antibodies still need to be resolved. Also, it will be essential to ultimately cross‐compare modes of action across species. Collective effort on such topics may be facilitated by recent initiatives aiming to bring together researchers specialised in different areas of milk related EV research, such as the task force on milk EVs initiated by the ISEV in 2022.
(3) While the influence of donor parameters are increasingly being recognised, it will be essential for the field to further map out the influence of donor‐to‐donor differences as well as inter‐donor alterations (in particular across lactation stage) on HMEV concentration, composition and activity. Our analysis further revealed that also much more basic aspects such as collection, storage and pre‐processing conditions of human milk, as well as the influence of pasteurisation, still need to be more systematically and comprehensively investigated. In fact, more systematic studies investigating the full impact of individual processing parameters starting from fresh milk without or with storage at different points during the sample handling procedure will be needed; This will provide an initial benchmark that future studies can then refer to and in particular facilitate the use of human milk biobanks; historically, the documentation and sample handling procedures of many human milk biobanks have typically been developed years ago without anticipating conditions and issues of relevance for HMEVs. A better knowledge of procedures compatible with HMEV isolation and retained quality, as well as relevant donor and sample data to be collected will create tremendous value for the use of prospectively generated human milk biobanks for HMEV research in the future. For clinical translation, a careful evaluation in particular of procedures which are compatible with clinical reality, ideally where mothers can collect the milk at home, will be key to eventually move the field towards therapeutic application of HMEVs.
(4) Another limiting factor for the field in developing both, a more comprehensive understanding of human milk EV biology as well as therapeutic HMEV applications is the characterisation in preclinical cellular or animal models, in particular under consideration of the following key aspects: *(i)* moving away from the reductionist approach to define the mode of action of HMEVs—just as for EVs in general—with a focus on individual molecules of interest (e.g. transfer of specific miRNAs) towards an unbiased, holistic assessment of cellular, tissue or organismal responses to HMEVs as a whole (e.g. multi ‐omics); *(ii)* thorough definition of dose‐responses with reporting of EV concentrations in particle numbers rather than total proteins, and detailed documentation of how concentrations were determined considering the substantial divergence between different platforms using even the same technology such as NTA (Figure 4C), *(iii)* transparent reporting of endotoxin content, in particular since HMEV activity is often studied in context with their anti‐inflammatory / immunomodulatory role, and *(iv)* rigorous inclusion of relevant controls, such as vesicles from other sources or EV depleted human milk samples to assess which activities are truly mediated by HMEVs versus co‐purifying components, as well as which activities are special for EVs from human milk versus other sources.

In summary, while human milk‐derived EVs may hold great promise as therapeutic agents, significant research will still be required to understand their full potential and limitations. Future studies should focus more on *(i)* systematically and comprehensively characterising HMEVs in relation to donor parameters, *(ii)* standardising protocols, improving analytical characterisation and documentation, and *(iii)* exploring and quantifying their activities in validated, translational preclinical models beyond rodents and with stringent controls. Additionally, substantial basic research is needed to better understand the molecular mechanism of action as well as to determine potentially relevant safety aspects to define critical quality attributes for each specific application.

## Author Contributions

The study was carried out by PH under guidance and supervision by NMK, with clinical input from DG and NH. The paper retrieval and further extraction strategy was defined by PH and GT under guidance by NMK. Retrieval and de‐duplication were performed by GT, extraction was performed by PH, PG and CTM, and consensus was reached after discussion with NMK. In‐depth analysis and discussion of all papers was performed by PH and NMK, with additional input on specific topics such as MISEV criteria (CTM, AM, MGim), therapeutic models (MG), translational issues (MGim, ER, DW, NH), human milk collection (MGs, DW, NH) and further refined by independent in‐depth assessment and input on the entire meta‐analysis and conclusions from MVW and MvH.

## Conflicts of Interest

The authors declare no conflicts of interest.

## Supporting information



Supporting Information: jev270287‐sup‐0001‐SuppMat.docx

Supporting Information: jev270287‐sup‐0002‐Table1.xlsx

Supporting Information: jev270287‐sup‐0003‐Table2.xlsx

Supporting Information: jev270287‐sup‐0004‐Fig4A.html

Supporting Information: jev270287‐sup‐0004‐Fig4B.html

Supporting Information: jev270287‐sup‐0004‐Fig4C.html

## Data Availability

Data sharing not applicable to this article as no datasets were generated or analysed during the current study.

## Notes

1 (Gao et al., 2019, Leiferman et al., 2019, Mirza et al., 2019, Reif et al., 2019, Vaswani et al., 2019, Wang et al., 2019, Bickmore and Miklavcic, 2020, Donalisio et al., 2020, Dong et al., 2020, Francese et al., 2020, Hu et al., 2020, Miyake et al., 2020, Reif et al., 2020, Zahoor et al., 2020, Bozack et al., 2021, Chen et al., 2021, Civra et al., 2021, Gutman‐Ido et al., 2021, He et al., 2021, He et al., 2021, Kupsco et al., 2021, Mourtzi et al., 2021, Pisano et al., 2020, Ramos‐Garcia et al., 2021, Shah et al., 2021, Vaswani et al., 2021, Wijenayake et al., 2021, Yun et al., 2021, Zhou et al., 2021, Zonneveld et al., 2021, Bozack et al., 2022, Chen et al., 2022, Cho et al., 2022, Guo et al., 2022, Hu et al., 2022, Karra et al., 2022, Kupsco et al., 2022, Ohta et al., 2022, Reif et al., 2022, Shah et al., 2022, Wang et al., 2022, Yan et al., 2022, Wang et al., 2022, Zhou et al., 2022, Azaryan et al., 2023, Chiba and Maeda, 2023, Freiria‐Martinez et al., 2023, Freiría‐Martínez et al., 2023, Giovanazzi et al., 2023, Gomez‐Ferrer et al., 2023, Hanna et al., 2023, Holzhausen et al., 2023, Holzhausen et al., 2023, Howe et al., 2023, Hu et al., 2023, Jiang et al., 2023, Kim et al., 2023, Meng et al., 2023, Ozturk et al., 2025, Shang et al., 2023, Tong et al., 2023, Torrez Lamberti et al., 2023, Turner et al., 2023, Vahkal et al., 2024, van Maanen et al., 2023, Wang et al., 2023, Weiskirchen et al., 2023, Zheng et al., 2023, Ahlberg et al., 2024, Albiach‐Delgado et al., 2024, Bonifay et al., 2024, Bozoglu et al., 2024, Cetinkaya et al., 2024, Gaylord et al., 2024, Holzhausen et al., 2024, Ma et al., 2024, Medel‐Martinez et al., 2024, Miklavcic et al., 2024, Muse et al., 2024, Vahkal et al., 2024, Wijenayake et al., 2024, Yung et al., 2024, Akinduro et al., 2025, Chutipongtanate et al., 2025, Golan‐Gerstl et al., 2025, Kim et al., 2025, Luo et al., 2025, Mo et al., 2025, Picans‐Leis et al., 2025, Sanjulian et al., 2025, Wang et al., 2025, Ye et al., 2025, Yuan et al., 2025)

2 (Leiferman et al., 2019, Bickmore and Miklavcic, 2020, Civra et al., 2021, Kupsco et al., 2021, Zonneveld et al., 2021, Cho et al., 2022, Karra et al., 2022, Kupsco et al., 2022, Freiria‐Martinez et al., 2023, Holzhausen et al., 2023, Howe et al., 2023, Kim et al., 2023, Meng et al., 2023, Turner et al., 2023, Vahkal et al., 2024, Weiskirchen et al., 2023, Ahlberg et al., 2024, Albiach‐Delgado et al., 2024, Cetinkaya et al., 2024, Gaylord et al., 2024, Miklavcic et al., 2024, Muse et al., 2024, Wijenayake et al., 2024, Yung et al., 2024, Chutipongtanate et al., 2025, Golan‐Gerstl et al., 2025, Kim et al., 2025, Ye et al., 2025, van Herwijnen et al., 2016, Blans et al., 2017, Vahkal et al., 2024)

3 (Liao et al., 2017, Miyake et al., 2020, Mirza et al., 2019, Vaswani et al., 2019, Wang et al., 2019, Donalisio et al., 2020, Francese et al., 2020, Chen et al., 2021, Gutman‐Ido et al., 2021, He et al., 2021, Mourtzi et al., 2021, Pisano et al., 2020, Ramos‐Garcia et al., 2021, Wijenayake et al., 2021, Zhou et al., 2021, Yan et al., 2022, Zhou et al., 2022, Giovanazzi et al., 2023, Gomez‐Ferrer et al., 2023, Hanna et al., 2023, Jiang et al., 2023, Tong et al., 2023, Torrez Lamberti et al., 2023, Wang et al., 2023, Zheng et al., 2023, Medel‐Martinez et al., 2024, Luo et al., 2025, Mo et al., 2025, Picans‐Leis et al., 2025, Wang et al., 2025, Yuan et al., 2025, Kahn et al., 2018, Freiria‐Martinez et al., 2023, Admyre et al., 2007, Batista et al., 2011, Lasser et al., 2011, Gutzeit et al., 2014, Zonneveld et al., 2014, Karlsson et al., 2016, Kosaka et al., 2010, Naftaly et al., 2025)

4 (Dong et al., 2020, Gao et al., 2019, Reif et al., 2019, Hu et al., 2020, Reif et al., 2020, Zahoor et al., 2020, Bozack et al., 2021, He et al., 2021, Shah et al., 2021, Vaswani et al., 2021, Yun et al., 2021, Bozack et al., 2022, Chen et al., 2022, Guo et al., 2022, Hu et al., 2022, Ohta et al., 2022, Reif et al., 2022, Shah et al., 2022, Wang et al., 2022, Wang et al., 2022, Azaryan et al., 2023, Chiba and Maeda, 2023, Hu et al., 2023, Ozturk et al., 2025, Shang et al., 2023, Bonifay et al., 2024, Bozoglu et al., 2024, Holzhausen et al., 2024, Ma et al., 2024, Akinduro et al., 2025, Sanjulian et al., 2025, Zhou et al., 2011, Naslund et al., 2014, Golan‐Gerstl et al., 2017, Larssen et al., 2017, Sims et al., 2017, Yang et al., 2017)

5 (Vaswani et al., 2019, Wang et al., 2019, Vaswani et al., 2021, Cetinkaya et al., 2024, Chutipongtanate et al., 2025, van Herwijnen et al., 2016, Blans et al., 2017, Admyre et al., 2007)

6 (He et al., 2021, Gao et al., 2019, Wang et al., 2019, Donalisio et al., 2020, Francese et al., 2020, Zahoor et al., 2020, Chen et al., 2021, Civra et al., 2021, Gutman‐Ido et al., 2021, Yun et al., 2021, Zhou et al., 2021, Ohta et al., 2022, Freiria‐Martinez et al., 2023, Freiría‐Martínez et al., 2023, Zheng et al., 2023, Yuan et al., 2025, Admyre et al., 2007, Gutzeit et al., 2014, Yang et al., 2017, He et al., 2021, Ye et al., 2025, Yan et al., 2022)

7 (He et al., 2021, Gao et al., 2019, Wang et al., 2019, Donalisio et al., 2020, Francese et al., 2020, Zahoor et al., 2020, Zahoor et al., 2020, Chen et al., 2021, Chen et al., 2021, Civra et al., 2021, Gutman‐Ido et al., 2021, Kupsco et al., 2021, Yun et al., 2021, Zhou et al., 2021, Ohta et al., 2022, Freiria‐Martinez et al., 2023, Freiría‐Martínez et al., 2023, van Maanen et al., 2023, Zheng et al., 2023, Bonifay et al., 2024, Yuan et al., 2025, Admyre et al., 2007, Gutzeit et al., 2014, Zhou et al., 2011, Yang et al., 2017, He et al., 2021, Ye et al., 2025)

8 (Dong et al., 2020, Vaswani et al., 2019, Reif et al., 2020, Vaswani et al., 2021, Chen et al., 2022, Guo et al., 2022, Guo et al., 2022, Chiba and Maeda, 2023, Ozturk et al., 2025, Shang et al., 2023, Torrez Lamberti et al., 2023, Weiskirchen et al., 2023, Bozoglu et al., 2024, Medel‐Martinez et al., 2024, Akinduro et al., 2025, Blans et al., 2017, Lasser et al., 2011, Naslund et al., 2014, Larssen et al., 2017, Martin et al., 2018, Hu et al., 2023, Kim et al., 2025, Ma et al., 2024, Wang et al., 2023)

9 (Miyake et al., 2020, Ramos‐Garcia et al., 2021, Wijenayake et al., 2021, Zhou et al., 2022, Wijenayake et al., 2024, Luo et al., 2025)

10 (Dong et al., 2020, Vaswani et al., 2019, Reif et al., 2020, Vaswani et al., 2021, Chen et al., 2022, Guo et al., 2022, Guo et al., 2022, Chiba and Maeda, 2023, Ozturk et al., 2025, Shang et al., 2023, Torrez Lamberti et al., 2023, Weiskirchen et al., 2023, Bozoglu et al., 2024, Medel‐Martinez et al., 2024, Akinduro et al., 2025, Blans et al., 2017, Lasser et al., 2011, Naslund et al., 2014, Larssen et al., 2017, Sims et al., 2017, Martin et al., 2018, Hu et al., 2023, Kim et al., 2025, Ma et al., 2024, Wang et al., 2023)

11 (Francese et al., 2020, Wijenayake et al., 2021, Freiria‐Martinez et al., 2023, Freiría‐Martínez et al., 2023, Turner et al., 2023, Batista et al., 2011, Lasser et al., 2011)

12 (Leiferman et al., 2019, Reif et al., 2019, Vaswani et al., 2019, Wang et al., 2019, Bickmore and Miklavcic, 2020, Bozack et al., 2021, Gutman‐Ido et al., 2021, Kupsco et al., 2021, Shah et al., 2021, Yun et al., 2021, Zhou et al., 2021, Bozack et al., 2022, Cho et al., 2022, Kupsco et al., 2022, Reif et al., 2022, Shah et al., 2022, Zhou et al., 2022, Holzhausen et al., 2023, Holzhausen et al., 2023, Holzhausen et al., 2023, Holzhausen et al., 2023, Howe et al., 2023, Kim et al., 2023, Ozturk et al., 2025, Zheng et al., 2023, Albiach‐Delgado et al., 2024, Bozoglu et al., 2024, Cetinkaya et al., 2024, Gaylord et al., 2024, Holzhausen et al., 2024, Wijenayake et al., 2024, Golan‐Gerstl et al., 2025, Picans‐Leis et al., 2025, Sanjulian et al., 2025, Blans et al., 2017, Kahn et al., 2018, Admyre et al., 2007, Karlsson et al., 2016, Kosaka et al., 2010, Golan‐Gerstl et al., 2017, He et al., 2021, Yan et al., 2022, Martin et al., 2018, Kim et al., 2025, Ma et al., 2024, Zahoor et al., 2020, Zhou et al., 2011, Hu et al., 2020, Jiang et al., 2023)

13 (He et al., 2021, Bozack et al., 2021, Bozack et al., 2022, Admyre et al., 2007, Gutzeit et al., 2014, Karlsson et al., 2016, Wang et al., 2021)

14 (Zahoor et al., 2020, Bozack et al., 2021, Mourtzi et al., 2021, Bozack et al., 2022, Karlsson et al., 2016, Naslund et al., 2014, Jiang et al., 2023)

15 (Miyake et al., 2020, Gao et al., 2019, Leiferman et al., 2019, Wang et al., 2019, Hu et al., 2020, Reif et al., 2020, Chen et al., 2021, Gutman‐Ido et al., 2021, He et al., 2021, Pisano et al., 2020, Ramos‐Garcia et al., 2021, Wijenayake et al., 2021, Yun et al., 2021, Zhou et al., 2021, Cho et al., 2022, Guo et al., 2022, Reif et al., 2022, Wang et al., 2022, Yan et al., 2022, Wang et al., 2022, Zhou et al., 2022, Gomez‐Ferrer et al., 2023, Hanna et al., 2023, Hu et al., 2023, Jiang et al., 2023, Meng et al., 2023, Ozturk et al., 2025, Tong et al., 2023, Torrez Lamberti et al., 2023, Vahkal et al., 2024, Weiskirchen et al., 2023, Zheng et al., 2023, Ahlberg et al., 2024, Albiach‐Delgado et al., 2024, Bozoglu et al., 2024, Ma et al., 2024, Medel‐Martinez et al., 2024, Wijenayake et al., 2024, Akinduro et al., 2025, Golan‐Gerstl et al., 2025, Luo et al., 2025, Mo et al., 2025, Wang et al., 2025, Ye et al., 2025, Admyre et al., 2007, Lasser et al., 2011, Naslund et al., 2014, Golan‐Gerstl et al., 2017, Larssen et al., 2017, Sims et al., 2017, Yang et al., 2017, Kim et al., 2025, Wang et al., 2023)

16 (Tran et al., 2025, Liao et al., 2017, Yanez‐Mo et al., 2015, Qian et al., 2023, Gao et al., 2019, Leiferman et al., 2019, Mirza et al., 2019, Wang et al., 2019, Bickmore and Miklavcic, 2020, Francese et al., 2020, Reif et al., 2020, Bozack et al., 2021, He et al., 2021, Kupsco et al., 2021, Shah et al., 2021, Bozack et al., 2022, Chen et al., 2022, Guo et al., 2022, Reif et al., 2022, Wang et al., 2022, Yan et al., 2022, Chiba and Maeda, 2023, Freiría‐Martínez et al., 2023, Zheng et al., 2023, Ahlberg et al., 2024, Bozoglu et al., 2024, Chutipongtanate et al., 2025, Luo et al., 2025, Ye et al., 2025, Vahkal et al., 2024, Admyre et al., 2007, Golan‐Gerstl et al., 2017)

17 (Yung et al., 2024, van Herwijnen et al., 2016, Admyre et al., 2007, Batista et al., 2011, Gutzeit et al., 2014, Zonneveld et al., 2014, Naslund et al., 2014)

18 (Vaswani et al., 2019, Vaswani et al., 2021, Kim et al., 2023, Turner et al., 2023, van Maanen et al., 2023, Blans et al., 2017, Kim et al., 2025, Hu et al., 2020, Chen et al., 2021, Chutipongtanate et al., 2022)

19 (Liao et al., 2017, Dong et al., 2020, Reif et al., 2019, Bickmore and Miklavcic, 2020, Donalisio et al., 2020, Francese et al., 2020, Civra et al., 2021, Shah et al., 2021, Wijenayake et al., 2021, Shah et al., 2022, Miklavcic et al., 2024, Kahn et al., 2018, Golan‐Gerstl et al., 2017, Zhou et al., 2011)

20 (Bozack et al., 2021, Kupsco et al., 2021, Bozack et al., 2022, Kupsco et al., 2022, Holzhausen et al., 2023, Holzhausen et al., 2023, Gaylord et al., 2024, Holzhausen et al., 2024, Karlsson et al., 2016)

21 (Mirza et al., 2019, Pisano et al., 2020, Vahkal et al., 2024, Weiskirchen et al., 2023, Albiach‐Delgado et al., 2024, Bozoglu et al., 2024, Ma et al., 2024)

22 (Tong et al., 2023, Ahlberg et al., 2024, Cetinkaya et al., 2024, Medel‐Martinez et al., 2024, Yung et al., 2024, Chutipongtanate et al., 2025, Luo et al., 2025, Wang et al., 2023)

23 (Miyake et al., 2020, Leiferman et al., 2019, Vaswani et al., 2021, Yun et al., 2021, Torrez Lamberti et al., 2023, Zheng et al., 2023, Lasser et al., 2011, Gutzeit et al., 2014, Yang et al., 2017, Yan et al., 2022, Hu et al., 2020, Jiang et al., 2023, Wang et al., 2021, Hu et al., 2022, Wang et al., 2022)

24
https://search.cosmobio.co.jp/view/p_view.asp?PrimaryKeyValue=19744966&ServerKey=&selPrice=1: Breast Milk exosome(CSR‐EXHM100L‐1SET[100uLx10 vial]) (Category: Proteins, Sugars, Hormones) translation from Japanese using GoogleTranslate).

25 (Leiferman et al., 2019, Ramos‐Garcia et al., 2021, Vaswani et al., 2021, Yun et al., 2021, Ohta et al., 2022, Wang et al., 2022, Freiria‐Martinez et al., 2023, Freiría‐Martínez et al., 2023, Giovanazzi et al., 2023, Gomez‐Ferrer et al., 2023, Hanna et al., 2023, Kim et al., 2023, Shang et al., 2023, Torrez Lamberti et al., 2023, Turner et al., 2023, van Maanen et al., 2023, Ahlberg et al., 2024, Albiach‐Delgado et al., 2024, Cetinkaya et al., 2024, Holzhausen et al., 2024, Ma et al., 2024, Miklavcic et al., 2024, Wang et al., 2025, Ye et al., 2025, van Herwijnen et al., 2016, Batista et al., 2011, Zonneveld et al., 2014, Karlsson et al., 2016, Kosaka et al., 2010, Larssen et al., 2017, Yang et al., 2017, Lukasik and Zielenkiewicz, 2014, Zhou et al., 2011, Chen et al., 2022)

26 (Ramos‐Garcia et al., 2021, Chen et al., 2022, Ohta et al., 2022, Torrez Lamberti et al., 2023, van Maanen et al., 2023, Larssen et al., 2017)

27 (Yun et al., 2021, Freiría‐Martínez et al., 2023, Hanna et al., 2023, Shang et al., 2023, Karlsson et al., 2016, Kosaka et al., 2010, Lukasik and Zielenkiewicz, 2014, Ma et al., 2024, Zhou et al., 2011, Wang et al., 2022)

28 (Leiferman et al., 2019, Ramos‐Garcia et al., 2021, Vaswani et al., 2021, Yun et al., 2021, Hanna et al., 2023, Kim et al., 2023, Shang et al., 2023, Torrez Lamberti et al., 2023, Ahlberg et al., 2024, Albiach‐Delgado et al., 2024, Wang et al., 2025, Larssen et al., 2017, Yang et al., 2017, Ye et al., 2025, Ma et al., 2024, Wang et al., 2023, Wang et al., 2022, Yun et al., 2020)

29 (Ohta et al., 2022, Freiria‐Martinez et al., 2023, Freiría‐Martínez et al., 2023, Gaylord et al., 2024, Holzhausen et al., 2024, Miklavcic et al., 2024, Karlsson et al., 2016, Zhou et al., 2011)

30 (Dong et al., 2020, Miyake et al., 2020, Leiferman et al., 2019, Mirza et al., 2019, Reif et al., 2019, Vaswani et al., 2019, Bickmore and Miklavcic, 2020, Donalisio et al., 2020, Francese et al., 2020, Reif et al., 2020, Civra et al., 2021, Gutman‐Ido et al., 2021, He et al., 2021, He et al., 2021, Kupsco et al., 2021, Pisano et al., 2020, Vaswani et al., 2021, Wijenayake et al., 2021, Zhou et al., 2021, Zonneveld et al., 2021, Karra et al., 2022, Kupsco et al., 2022, Wang et al., 2022, Yan et al., 2022, Wang et al., 2022, Zhou et al., 2022, Giovanazzi et al., 2023, Gomez‐Ferrer et al., 2023, Howe et al., 2023, Kim et al., 2023, Meng et al., 2023, Ozturk et al., 2025, Tong et al., 2023, Torrez Lamberti et al., 2023, Turner et al., 2023, Wang et al., 2023, Weiskirchen et al., 2023, Zheng et al., 2023, Ahlberg et al., 2024, Albiach‐Delgado et al., 2024, Cetinkaya et al., 2024, Medel‐Martinez et al., 2024, Miklavcic et al., 2024, Muse et al., 2024, Wijenayake et al., 2024, Akinduro et al., 2025, Chutipongtanate et al., 2025, Golan‐Gerstl et al., 2025, Luo et al., 2025, Mo et al., 2025, Picans‐Leis et al., 2025, Wang et al., 2025, Blans et al., 2017, Karlsson et al., 2016, Sims et al., 2017, Martin et al., 2018, Kim et al., 2025, Jiang et al., 2023, Holzhausen et al., 2023, Chen et al., 2021, Guo et al., 2022)

31 (Ramos‐Garcia et al., 2021, Gomez‐Ferrer et al., 2023, Hu et al., 2023, Torrez Lamberti et al., 2023, Ye et al., 2025, Wang et al., 2019)

32 (Mirza et al., 2019, Vaswani et al., 2019, Pisano et al., 2020, Azaryan et al., 2023, Shang et al., 2023, Turner et al., 2023, Golan‐Gerstl et al., 2025, Yuan et al., 2025, Admyre et al., 2007)

33 (Pisano et al., 2020, Vaswani et al., 2021, Ohta et al., 2022, Torrez Lamberti et al., 2023, Turner et al., 2023, Gutzeit et al., 2014, Yan et al., 2022, Wang et al., 2022)

34 (Vaswani et al., 2021, Wang et al., 2022, Freiría‐Martínez et al., 2023, Kim et al., 2023, Gaylord et al., 2024, Ma et al., 2024, Karlsson et al., 2016, Kosaka et al., 2010, Lukasik and Zielenkiewicz, 2014, Zhou et al., 2011, Yun et al., 2020)

35 (Mirza et al., 2019, Bozack et al., 2021, Gutman‐Ido et al., 2021, Kupsco et al., 2021, Mourtzi et al., 2021, Shah et al., 2021, Bozack et al., 2022, Cho et al., 2022, Kupsco et al., 2022, Shah et al., 2022, Holzhausen et al., 2023, Holzhausen et al., 2023, Howe et al., 2023, Vahkal et al., 2024, Zheng et al., 2023, Muse et al., 2024, Chutipongtanate et al., 2025, Golan‐Gerstl et al., 2025, Picans‐Leis et al., 2025, Sanjulian et al., 2025, Gutzeit et al., 2014, Zahoor et al., 2020)

36 (Mirza et al., 2019, Bozack et al., 2021, Gutman‐Ido et al., 2021, Kupsco et al., 2021, Shah et al., 2021, Bozack et al., 2022, Cho et al., 2022, Kupsco et al., 2022, Shah et al., 2022, Holzhausen et al., 2023, Holzhausen et al., 2023, Howe et al., 2023, Zheng et al., 2023, Muse et al., 2024, Sanjulian et al., 2025, Gutzeit et al., 2014, Zahoor et al., 2020)

37 (Zahoor et al., 2020, Bozack et al., 2021, Kupsco et al., 2021, Mourtzi et al., 2021, Shah et al., 2021, Bozack et al., 2022, Kupsco et al., 2022, Shah et al., 2022, Holzhausen et al., 2023, Howe et al., 2023, Muse et al., 2024, Picans‐Leis et al., 2025, Sanjulian et al., 2025, Holzhausen et al., 2023)

38 (Mirza et al., 2019, Vahkal et al., 2024, Zheng et al., 2023, Golan‐Gerstl et al., 2025, Gutzeit et al., 2014, Cho et al., 2022)

39 (Liao et al., 2017, Dong et al., 2020, Miyake et al., 2020, Gao et al., 2019, Wang et al., 2019, Donalisio et al., 2020, Francese et al., 2020, Reif et al., 2020, Civra et al., 2021, Gutman‐Ido et al., 2021, He et al., 2021, Pisano et al., 2020, Zonneveld et al., 2021, Karra et al., 2022, Reif et al., 2022, Zhou et al., 2022, Azaryan et al., 2023, Chiba and Maeda, 2023, Meng et al., 2023, Tong et al., 2023, Bonifay et al., 2024, Bozoglu et al., 2024, Yung et al., 2024, Kahn et al., 2018, Admyre et al., 2007, Lasser et al., 2011, Naslund et al., 2014, Golan‐Gerstl et al., 2017, Yan et al., 2022, Martin et al., 2018, Hu et al., 2023, Hu et al., 2020, Jiang et al., 2023, Chen et al., 2021, Hu et al., 2022, Wang et al., 2022, Guo et al., 2022, Zhou et al., 2022, He et al., 2021)

40 (Dong et al., 2020, Gao et al., 2019, Wang et al., 2019, Donalisio et al., 2020, Francese et al., 2020, Reif et al., 2020, Civra et al., 2021, Pisano et al., 2020, Reif et al., 2022, Ozturk et al., 2025, Vahkal et al., 2024, Naslund et al., 2014, Chen et al., 2021, Guo et al., 2022, Arroyo et al., 2011, He et al., 2021, He et al., 2021, Martin et al., 2018, Miyake et al., 2020, Wang et al., 2022, Zhou et al., 2022, Karra et al., 2022, Bozoglu et al., 2024, Akinduro et al., 2025, Azaryan et al., 2023, Kim et al., 2023)

41 (Miyake et al., 2020, Gao et al., 2019, Gutman‐Ido et al., 2021, Pisano et al., 2020, Zhou et al., 2021, Reif et al., 2022, Wang et al., 2022, Zhou et al., 2022, Hu et al., 2023, Meng et al., 2023, Ozturk et al., 2025, Tong et al., 2023, Vahkal et al., 2024, Bozoglu et al., 2024, Wijenayake et al., 2024, Akinduro et al., 2025, Luo et al., 2025, Mo et al., 2025, Admyre et al., 2007, Yan et al., 2022, Martin et al., 2018, Zhou et al., 2011, Jiang et al., 2023, Chen et al., 2021, Hu et al., 2022, Guo et al., 2022, Wang et al., 2019)

42 (Liao et al., 2017, Dong et al., 2020, Reif et al., 2019, Donalisio et al., 2020, Francese et al., 2020, Reif et al., 2020, Civra et al., 2021, Kahn et al., 2018, Golan‐Gerstl et al., 2017)

43 (He et al., 2021, Dong et al., 2020, Miyake et al., 2020, Gao et al., 2019, Wang et al., 2019, Pisano et al., 2020, Guo et al., 2022, Vahkal et al., 2024, Yuan et al., 2025, Yan et al., 2022, Martin et al., 2018, Hu et al., 2023, Chen et al., 2021)

44 (Dong et al., 2020, Miyake et al., 2020, Gao et al., 2019, Zhou et al., 2021, Karra et al., 2022, Zhou et al., 2022, Azaryan et al., 2023, Wijenayake et al., 2024, Akinduro et al., 2025, Martin et al., 2018, Guo et al., 2022, Wang et al., 2019)

45 (He et al., 2021, Pisano et al., 2020, Ozturk et al., 2025, Vahkal et al., 2024, Naslund et al., 2014, Kim et al., 2025, Guo et al., 2022)

46 (Bickmore and Miklavcic, 2020, Kupsco et al., 2021, Zonneveld et al., 2021, Giovanazzi et al., 2023, Gomez‐Ferrer et al., 2023, Turner et al., 2023, van Herwijnen et al., 2016, Batista et al., 2011, Lasser et al., 2011, Gutzeit et al., 2014, Zonneveld et al., 2014, Zhou et al., 2011, Naslund et al., 2014, Martin et al., 2018, Holzhausen et al., 2023)
