# Supplementary material for: Current Perspective on Human Milk Derived Vesicles and Their Potential Therapeutic Use: A Scoping Review
Source: J Extracell Vesicles. 2026 May 15;15:e70287. doi: 10.1002/jev2.70287 (PMC13178797; doi:10.1002/jev2.70287)
Supplement: Supplementary file 1 — Supporting Information: jev270287‐sup‐0001‐SuppMat.docx [file JEV2-15-e70287-s006.docx]

**Search strategy**

Following the guidelines described in Preferred Reporting Items for Systematic reviews and Meta-Analyses for Scoping reviews (PRISMA-ScR) we completed the search by employing a previously published strategy by O’Reilly et al. (2019). For our search, we used the following key terms to extract articles from the databases Embase and MEDLINE on human milk extracellular vesicles. The search was completed on 27^th^ May 2025.

**Embase + Classic Embase**

1 *breast milk* or *breastmilk* or *breast milks* or *human milk* or *human breast milk* or *breast milk human* or *mature milk* or *colostrum* or *early milk* or *transitional milk* .ab,kw,ti.

2 (*breastfeeding* or *breast feeding* or *breast-feeding*).ab,kw,ti.

3 *breast milk*/

4 *breast feeding*/ or *lactation*/

5 1 or 2 or 3 or 4

6 *extracellular vesicle** or *extracellular membrane vesicle** or *membrane vesicle** or *microparticle** or *microvesicle** or *exosome** or *ectosome** or *shedding vesicle** or *membrane particle** or *secretory vesicle** or *cell-derived microparticle** or *nanovesicle** or *exovesicle** or *tolerosome** or *shedding microvesicle**).ab,kw,ti.

7 *exosome*/

8 *membrane vesicle*/

9 *nanoparticle*/

10 6 or 7 or 8 or 9

11 5 and 10

**Ovid Medline(r)**

1 (*breast milk* or *breastmilk* or *breast milks* or *human milk* or *human breast milk* or *breast milk human* or *mature milk* or *colostrum* or *early milk* or *transitional milk*).ab,kf,ti.

2 (*breastfeeding* or *breast feeding* or *breast-feeding*).ab,kf,ti.

3 *milk, human*/

4 *breast feeding*/

5  *lactation/*/

6 1 or 2 or 3 or 4 or 5

7 (*extracellular vesicle** or *extracellular membrane vesicle** or *membrane vesicle** or *microparticle** or *microvesicle** or *exosome** or *ectosome** or *shedding vesicle** or *membrane particle** or *secretory vesicle** or *cell-derived microparticle** or *nanovesicle** or *exovesicle** or *tolerosome** or *shedding microvesicle**).ab,kf,ti.

8 *nanoparticles*/ or *exosomes*/

9 7 or 8

10 6 and 9

**Trial registries**

To identify any clinical trials on human milk EVs for inclusion in this Scoping Review, we performed a search of three trial registries: ClinicalTrials.gov, International Clinical Trials Registry Platform (ICTRP) and European Union Drug Regulating Authorities Clinical Trials platform (EudraCT). The initial search involved the loose key terms ‘extracellular vesicle(s)’ and ‘exosome(s)’, to avoid missing relevant trials due too narrow definition of the search words. Search results were screened independently by two reviewers to identify trials involving human milk extracellular vesicles and possible disputes were resolved upon discussion. When screening the trial registries, we focused on clinical trials including human milk EV application for interventional studies. Therefore, we excluded studies using human milk EVs as a disease marker, not a treatment.

All extracted articles were inspected manually to remove any additional duplicates and verify that they met all the above-mentioned search criteria and contain: (1) **original data published in peer-reviewed journals and (2) studies of extracellular vesicles from human milk.** We excluded reviews, editorials, letters to the editor, conference abstracts and case reports. For the meta-analysis provided in the article, titles, abstracts and texts were then fully assessed by 2 reviewers independently. In case of a possible disagreement, consensus was reached upon discussion with a third expert.
